# Characterizing the composition and diversity of the vaginal microbiome in ovarian cancer

**DOI:** 10.1038/s43856-026-01771-8

**Published:** 2026-07-10

**Authors:** April Deveaux, Oyomoare L. Osazuwa-Peters, Yeon Ji Kim, Pixu Shi, Drew Neish, Ashwini Joshi, Veronica Duck, Ashliegh Williams, Margaret Gates Kuliszewski, Bin Huang, Kevin Ward, Maria Pisu, Thomas Tucker, Rebecca Previs, Andrew Berchuck, Tomi Akinyemiju

**Affiliations:** 1 Department of Population Health Sciences, Duke University School of Medicine, Durham, NC USA; 2 Duke Cancer Institute, Durham, NC USA; 3 Microbiome Core Facility, Department of Molecular Genetics and Microbiology, Duke University School of Medicine, Durham, NC USA; 4 Biostatistics & Bioinformatics, Duke University School of Medicine, Durham, NC USA; 5 New York State Cancer Registry, New York State Department of Health, Albany, NY USA; 6 Kentucky Cancer Registry, University of Kentucky, Lexington, KY USA; 7 University of Kentucky, Lexington, KY USA; 8 Georgia Cancer Registry, Emory University, Atlanta, GA USA; 9 Division of Preventive Medicine and O’Neal Comprehensive Cancer Center, University of Alabama at Birmingham, Birmingham, AL USA; 10 Department of Epidemiology and Environmental Health, University of Kentucky, Lexington, KY USA; 11 Labcorp Oncology, Durham, NC USA; 12 Duke Division of Gynecologic Oncology, Durham, NC USA

**Keywords:** Ovarian cancer, Epidemiology

## Abstract

**Background:**

Vaginal dysbiosis, characterized by *Lactobacillus* depletion and anaerobic enrichment, may be relevant to ovarian cancer (OC) outcomes, yet comprehensive microbiome characterization in OC patients remains limited. Here, we characterize the prevalence and predictors of vaginal microbiome dysbiosis among racially diverse OC patients in the US.

**Methods:**

We performed 16S rRNA gene sequencing on vaginal samples from 132 OC patients recruited as part of the population-based ORCHiD study. Latent Dirichlet Allocation (LDA), a computational topic modeling approach, was applied to identify distinct microbial community signatures representing co-occurring bacterial taxa.

**Results:**

Here, we show that *Lactobacillus* is detected in only 47.7% of patients. Topic modeling identifies seven distinct microbial signatures spanning *Lactobacillus*-dominated and anaerobe-enriched community types. Anaerobic bacterial enrichment (*Peptoniphilus*, *Anaerococcus*) increases significantly with age (approximately 35% per decade of life, FDR q = 0.019). Racial differences are observed, with non-Hispanic Black patients demonstrating a 5-fold higher prevalence of an Actinomycetaceae-classified amplicon sequence variant (ASV54) compared with non-Hispanic white patients (40.9% vs 8.2%, FDR q = 0.005), while *Lactobacillus crispatus* is detected only among non-Hispanic white patients (6.4% vs 0%) in this cohort.

**Conclusions:**

These findings demonstrate a high burden of vaginal microbiome dysbiosis among OC patients and identify age- and race-associated microbial patterns that may be relevant to understanding disparities in OC outcomes.

## Plain language summary

Ovarian cancer affects thousands of women each year and has poor survival rates. The vaginal microbiome, the community of bacteria in the vagina, may play a role in cancer outcomes. Healthy vaginas typically contain a beneficial species of bacteria called *Lactobacillus*, but when these decline, harmful bacteria can increase, causing inflammation. We studied vaginal bacteria in 132 women with ovarian cancer and found that less than half had healthy levels of *Lactobacillus*. Older women were more likely to have harmful kinds of bacteria, and Black and white women showed different bacterial patterns. These differences in vaginal bacteria might help explain why some women have worse cancer outcomes. Understanding these patterns could lead to new ways to improve treatment and outcomes for women with ovarian cancer.

## Abstract (web summary)

Deveaux et al. characterize the vaginal microbiome in 132 ovarian cancer patients using 16S rRNA sequencing and topic modeling. Widespread *Lactobacillus* depletion, age-associated anaerobic dysbiosis, and racial differences in microbial composition are identified that may contribute to disparities in ovarian cancer outcomes.

## Introduction

The vaginal microbiome, the community of microbes in the lower female genital tract, has emerged as an important factor in gynecologic health and gynecologic cancers and may influence disease prognosis^1,2^. A healthy microbiome is typically dominated by *Lactobacillus* species, which help maintain a low pH and protect against pathogens^3,4^. When *Lactobacillus* is no longer dominant—a state known as vaginal dysbiosis—the microbiome shifts toward communities dominated by anaerobic bacteria^4^. This dysbiotic state has been linked to adverse vaginal health outcomes such as bacterial vaginosis, pelvic inflammatory disease and chronic genital inflammation^2,5,6^, and can contribute to immune dysregulation, epithelial dysfunction, and pro-inflammatory signaling—important mechanisms in tumor progression^5,7^.

Ovarian cancer (OC) remains one of the most lethal gynecologic malignancies in the United States, with a 5-year survival rate of only 51%^8^; however, few studies have characterized the role of the vaginal microbiome in contributing to OC prognosis. Recent studies suggest that OC is associated with a shift in the vaginal microbiome toward a dysbiotic state^3,5,9^. In a case-control study with ovarian cancer patients, Morikawa et al. found that the cervicovaginal microbiota of OC patients was characterized by significant loss of *Lactobacillus*-dominated communities and increased microbial diversity across multiple histologic subtypes compared to healthy controls^10^. Sipos et al. proposed the concept of an “oncobiome,” describing how pathogen-driven disruptions, such as infections with *Chlamydia trachomatis* or *Neisseria gonorrhoeae*, may lead to lasting alterations in the vaginal microbiome that promote chronic inflammation and carcinogenesis^11^. Building on this, Mehra et al. reviewed evidence of altered vaginal and gut microbial ecosystems in OC, reporting that *Lactobacillus* was present in only 24% of women with OC compared to 47% of healthy controls^12^. They also identified dysbiosis-associated microbial shifts as contributors to immune dysregulation and tumor progression, suggesting the potential for microbiome modulation as a diagnostic or therapeutic strategy^12^. Together, these findings describe a broader biological framework in which vaginal dysbiosis is both a marker and a possible mediator of OC pathophysiology.

The vaginal microbiome varies by patient demographics and clinical factors^13,14^. For instance, aging is associated with declining estrogen levels and shifts in immune function that reduce *Lactobacillus* abundance while increasing anaerobic bacterial diversity^15^. The vaginal microbiome composition also varies by race/ethnicity, with patterns most extensively characterized in U.S.-based cohorts^16^, including documented differences in microbial diversity, bacterial species composition, and *Lactobacillus* distribution patterns^17–20^. Such compositional differences might contribute to disparities in OC outcomes, including tumor aggressiveness and progression^5,21,22^. Notably, Black women experience significantly worse OC outcomes compared to white women, including later-stage diagnosis, more aggressive tumor biology, and reduced survival^23,24^. While socioeconomic factors and healthcare access are major drivers of these disparities, differences in microbial profiles may reflect biologic embedding of social and structural inequities (e.g., differential exposures, healthcare access, and treatment experiences)^25^, and could be associated with downstream pathways relevant to OC outcomes^19,26^. A modest number of prior studies have investigated the role of the vaginal microbiome in OC outcomes^3,27–29^; however, these investigations have largely evaluated alpha and beta diversity, differential abundance testing, and taxonomic profiling based on relative abundances^30,31^. These traditional analytical approaches, while informative, are unable to identify complex community-level patterns that emerge from co-occurring bacterial taxa^31,32^, insights that might reveal new mechanistic pathways contributing to OC outcomes and disparities.

Here, we characterize vaginal microbiome composition, diversity, and dysbiosis among ovarian cancer patients in the population-based ORCHiD study. We identify widespread *Lactobacillus* depletion and distinct microbial community signatures, with significant age-associated anaerobic enrichment and marked racial differences in microbial composition. These findings provide insight into microbiome-associated patterns that may contribute to disparities in OC outcomes.

## Methods

### Study design and population

**Fig. 1:**
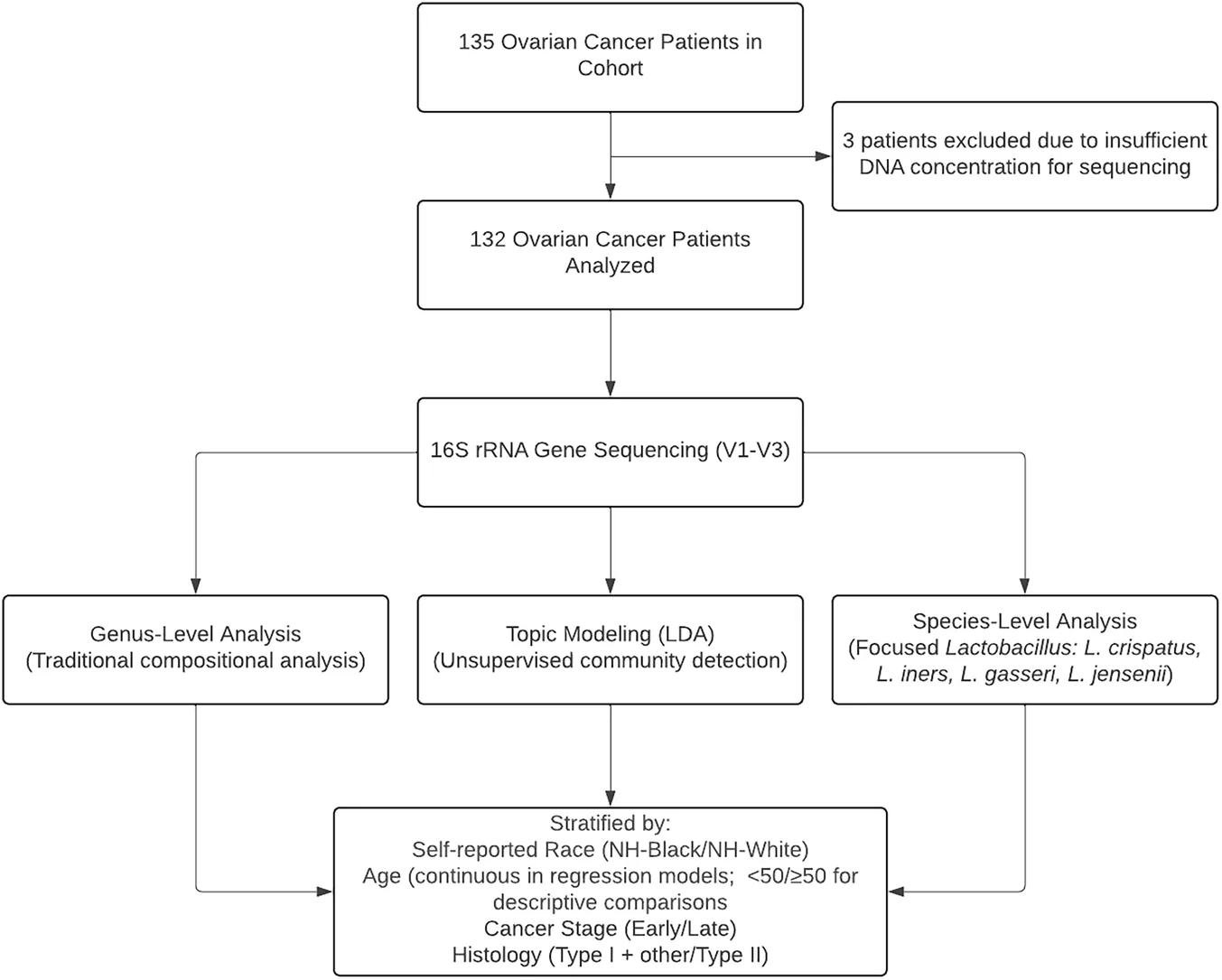
Study design and multi-scale analytical framework for dysbiotic populations. Flowchart illustrating the analytical approach used in the ORCHiD study. A total of 135 OC patients provided vaginal samples for 16S rRNA gene sequencing (V1-V3 region). Three were excluded due to insufficient DNA quantity. Three complementary analytical approaches were employed: traditional genus-level analysis, topic modeling using Latent Dirichlet Allocation (LDA) for unsupervised community detection, and species-level analysis focused on *L. crispatus*, *L. iners*, *L. gasseri*, and *L. jensenii*. All analyses were stratified by self-reported race (NH-Black/NH-white), age (continuous for regression analysis, categorical (<50/≥50 years) for descriptive comparisons), cancer stage (Early/Late), and histology (Type I epithelial/+other/Type II epithelial).

An overview of the study design and framework is presented in Fig. 1. This study included 132 ovarian cancer (OC) patients recruited as part of the ORCHiD study. Detailed methods are published elsewhere^33^. Briefly, this population-based study recruited patients with confirmed first primary OC between March 2021 and October 2024 from seven state cancer registries, including New York, Kentucky, California, North Carolina, Maryland, Georgia, and Texas. The study inclusion criteria were patients aged 18 years and older with a pathologically confirmed diagnosis of OC (FIGO stages I-IV), approximately 9–12 months prior to recruitment. All patients in this cohort self-identified their race/ethnicity as non-Hispanic Black (NH-Black) or non-Hispanic white (NH-white). Participants from other racial/ethnic groups were not included in the study due to insufficient sample size. The study exclusion criteria were patients older than 79 years or patients with cognitive impairments preventing survey completion. The ORCHiD survey comprehensively captures data on patient demographics, socioeconomic status, healthcare access, as well as self-reported treatment, lifestyle, and health history data. Eligible patients were contacted via phone to confirm eligibility, conduct informed consent, and determine participation interest. Patients who consent to participate complete the ORCHiD survey over the phone, online, or by mail and receive an incentive of $25 for survey completion.

### Vaginal microbiome sample collection and processing

Patients who completed the ORCHiD survey were offered an opportunity to participate in the ORCHiD biospecimen substudy, which required self-collection of both a vaginal swab and saliva sample. Once a patient consented to the substudy, the study team mailed a biospecimen packet containing a vaginal microbiome collection kit (OMR-130, DNA Genotek, Ottawa, Canada), manufacturer instructions and a postage-paid sample return envelope. Each biospecimen packet also included a brief four-question behavioral survey querying medications or microbiome-altering behaviors in the previous 30 days, including oral or intravenous antibiotic use, vaginal douching, or use of intravaginal suppository products. Patients responding yes to any of the microbiome survey questions were excluded from the analysis cohort. Patients were also instructed not to collect samples during active menstruation or other vaginal bleeding. Upon receipt of returned kits, samples were inspected for any damage or leakage and logged electronically. The collection kit included a swab and a vial containing microbial media buffer, a proprietary stabilizing solution to preserve samples at ambient temperature for up to 30 days. Any damaged kits were disposed of according to established safety regulations. Because samples were self-collected and returned by mail, there was variability in the timing of sample receipt following collection. According to manufacturer specifications, samples preserved in the OMNIgene vaginal collection kit are stable at ambient temperature for up to 30 days. Upon receipt at the study coordinating center, samples were logged and transferred for processing, with efforts made to initiate processing within approximately 3 weeks of receipt when feasible. Samples were processed by treating with 5 μl of Proteinase K (80 mg/ml) followed by incubation for 1 h in a 50 °C water bath. Samples were then aliquoted into 1.5 ml microcentrifuge tubes and stored at −80 °C until transfer to the Duke Microbiome Core Facility for DNA extraction and sequencing. Due to batch-based processing and sequencing workflows, the duration of −80 °C storage varied across samples but remained within standard laboratory practices for long-term microbiome sample preservation. Patients who completed and returned the biospecimen kits received an additional $20 incentive.

Microbial DNA from vaginal swab samples preserved in OMNIgene vaginal kits was extracted using the DNeasy 96 PowerSoil Pro QIACube HT kit (QIAGEN, #47021) on an automated QIACube HT platform (QIAGEN). The manufacturer’s instructions were followed with these minor deviations: 250 µl of the preserved sample was used as starting material, and during the final elution step, the elution buffer (C6) was incubated at room temperature for 10 min to increase DNA yield. DNA was eluted in a final volume of 100 µl. Bacterial community composition was characterized by amplification of the 16S rRNA V1-V3 hypervariable regions via polymerase chain reaction (PCR). Forward primer 27F (5’-GAGTTTGATCGTGGCTCAG-3’) and reverse primer 518R (5’-ATTACCGCGGCTGCTGG-3’) were used with Phusion Plus PCR Master Mix (ThermoScientific, #F631L) for a total of 25 PCR cycles (initial denaturation at 98 °C, annealing at 58 °C, extension at 72 °C). Samples were multiplexed using reverse primers (518R) that carry unique Earth Microbiome Project (http://www.earthmicrobiome.org/) barcodes^34^. PCR products were purified with AMPure XP Beads (Beckman Coulter, #A63881) and quantified with a Qubit dsDNA HS assay kit (ThermoFisher, #Q32854) on a Promega GloMax plate reader. Equimolar PCR products were pooled from each sample and submitted to the Duke Sequencing and Genomic Technologies shared resource. The final pool was sequenced on an Illumina MiSeq platform using v3 chemistry (300 base pairs, paired-end).

### Study variables

Cohort characteristics assessed included self-reported race, age at diagnosis, OC stage, and histologic subtype. Race was self-reported and categorized as non-Hispanic Black or non-Hispanic white. Age at diagnosis was analyzed as a continuous variable in all multivariable models to maximize statistical power and capture gradual age-related changes in microbiome composition. For descriptive summaries and comparison with prior literature, age is reported categorically (<50 years and ≥50 years) where appropriate. Clinical data, such as stage at diagnosis and histological subtype, were obtained from state cancer registries. OC stage was derived from summary staging data and categorized into early-stage (summary stage 1 or 2) or late-stage (summary stage ≥3). Histologic subtype was categorized as either type II epithelial (including high-grade serous and other aggressive subtypes) or non-type II (including type I epithelial, endometrioid, mucinous, clear cell subtypes, and non-epithelial tumors).

Covariates included in multivariable models were selected a priori based on established associations with vaginal microbiome composition and ovarian cancer characteristics. Race, age at diagnosis, disease stage, and histologic subtype were included to account for demographic, clinical, and tumor-related factors that may influence microbial composition and confound observed associations. Socioeconomic variables, including education and income, were collected and are reported descriptively (Table 1). However, these variables were not included in the primary multivariable models for microbiome analyses. This decision was based on several considerations^1^: the high dimensionality of genus-level microbiome data (239 genera) relative to sample size (*N* = 132) would require extensive multiple testing correction, substantially reducing statistical power to detect true associations^2^; the addition of multiple socioeconomic covariates to high-dimensional models risks overfitting and unstable parameter estimates. The topic-level framework established in this study (reducing 239 genera to 7 biologically meaningful topics) provides a foundation for future socioeconomic analyses with improved statistical efficiency in larger cohorts.

**Table 1:** Baseline characteristics of ovarian cancer patients in the ORCHiD study cohort

	Overall (%)	NH-Black (%)	NH-white (%)
	(*N* = 132)	(*N* = 22)	(*N* = 110)
Age group			
<50	22 (16.7)	5 (22.7)	17 (15.5)
50+	110 (83.3)	17 (77.3)	93 (84.5)
Region			
Midwest	20 (15.2)	1 (4.5)	19 (17.3)
Northeast	34 (25.8)	9 (40.9)	25 (22.7)
South	47 (35.6)	11 (50.0)	36 (32.7)
West	31 (23.5)	1 (4.5)	30 (27.3)
Stage			
Early	41 (31.1)	5 (22.7)	36 (32.7)
Late	91 (68.9)	17 (77.3)	74 (67.3)
Histology group			
Type I epithelial	42 (31.8)	6 (27.3)	36 (32.7)
Type II epithelial	80 (60.6)	13 (59.1)	67 (60.9)
Other	10 (7.6)	3 (13.6)	7 (6.4)
Income			
Less than $20,000	13 (9.6)	5 (22.7)	8 (7.3)
At least $20,000 but less than $40,000	17 (12.9)	6 (27.3)	11 (10.0)
At least $40,000 but less than $60,000	23 (17.4)	5 (22.7)	18 (16.4)
At least $60,000 but less than $80,000	12 (9.1)	0 (0)	12 (10.9)
More than $80,000	64 (48.5)	6 (27.3)	58 (52.7)
Missing	3 (2.3)	0 (0)	3 (2.7)
Employment status			
Disabled	9 (6.8)	6 (27.3)	3 (2.7)
Employed full or part-time	54 (40.9)	7 (31.8)	47 (42.7)
Other	4 (3.0)	1 (4.5)	3 (2.7)
Retired	58 (43.9)	5 (22.7)	53 (48.2)
Unemployed	6 (4.5)	3 (13.6)	3 (2.7)
Missing	1 (0.8)	0 (0)	1 (0.9)
Education			
High school graduate or GED	17 (12.9)	4 (18.2)	13 (11.8)
Some college or 2-year degree	34 (25.8)	5 (22.7)	29 (26.4)
4-year college graduate	39 (29.5)	5 (22.7)	34 (30.9)
More than 4-year college degree	42 (31.8)	8 (36.4)	34 (30.9)
Marital status			
Married/living with partner	79 (59.8)	5 (22.7)	74 (67.3)
Widowed/divorced/separated/never married	52 (39.4)	16 (72.7)	36 (32.7)

### Statistical analysis

#### Data processing

Microbiome sequencing data were processed using the R programming suite (v4.4.0)^35^. Sequencing reads from vaginal samples were processed using the DADA2 pipeline (v1.28.0)^36^ to infer amplicon sequence variants (ASVs) with default parameters. Deviations from default parameters include a parameter for FilterAndTrim function (truncLen = c(290, 260)) to remove sequencing regions with low quality scores and only including ASVs that were 433 to 525 base pairs long in analysis. Taxonomy was assigned using the Silva v138.1 database^37,38^ and non-bacterial taxa were removed. While specialized vaginal microbiome databases (e.g., SpeciateIT/vSpeciateDB^39^) offer enhanced species-level resolution for select vaginal taxa, Silva provides comprehensive taxonomic coverage suitable for genus-level community analyses. Therefore, species-level taxonomic inference in this study was restricted to targeted analyses of *Lactobacillus*, where Silva performs adequately for clinically relevant species discrimination. Samples with less than 1000 reads were removed, which included four negative controls and three samples with insufficient DNA quantity, and a phylogenetic tree was constructed using Randomized Axelerated Maximum Likelihood (RAxML) to account for phylogenetic relationships^40^.

#### Operational definition of vaginal microbiome dysbiosis

Vaginal microbiome dysbiosis was operationally defined as non–*Lactobacillus*-dominant communities, where *Lactobacillus* comprised <50% relative abundance of the vaginal microbiome. *Lactobacillus* dominance (≥50% relative abundance) was used as a reference state for comparison. This threshold was applied for analytic consistency and does not imply a clinical diagnosis. Dysbiosis was further characterized using taxon-level abundance metrics and topic model–derived community signatures to capture heterogeneity among non–*Lactobacillus*-dominant profiles.

#### Summary statistics

For taxonomic composition analysis, we calculated three key metrics for each bacterial genus^1^: prevalence, defined as the percentage of samples in which the genus was detected above the minimum threshold^2^; mean relative abundance, calculated as the average percentage of total reads attributed to each genus across all samples; and^3^ median relative abundance with interquartile range (IQR), providing robust measures of central tendency and spread for non-normally distributed abundance data. A genus was considered ‘detected’ if it had ≥10 reads in a sample (or ≥0.01% relative abundance).

#### Topic modeling analysis

We employed Latent Dirichlet Allocation (LDA) to identify distinct vaginal microbial community signatures, termed “microbial topics,” representing co-occurring bacterial taxa^41^, as previously proposed for microbiome data^42^ and applied in vaginal microbiome studies^43^. The range of 3 to 10 topics was selected based on established vaginal microbiome community classifications, as the foundational community state type (CST) framework identifies 5 CSTs with up to 9 CSTs with extended classification^16,44^. This biologically informed range was selected to align with known vaginal microbiome structure while allowing for community patterns not previously described in this cancer population. Optimal topic number was determined using the FindTopicsNumber function from the “ldatuning” R package (v1.0.2)^45^ with multiple metrics, including CaoJuan2009, Arun2010, Griffiths2004, and Deveaud2014^46–49^, evaluated across the topic number range. Two metrics (CaoJuan2009 and Arun2010) identify optimal models by minimization, while the other two metrics (Griffiths2004 and Deveaud2014) identify optimal models by maximization. The optimal number of topics was selected using an elbow method approach (Supplemental Fig. S1). LDA was fitted to the data using the “topicmodels” package (v0.2.17) in R^50^ with Gibbs sampling method, treating samples as “documents” and genera as “words.” The model was run with a fixed seed (243) for reproducibility. Beta probability matrices (genus-topic associations) and gamma probability matrices (sample-topic associations) were extracted using the “tidytext” package (v0.4.3)^51^. To assess robustness of the topic number^52^, model stability was evaluated across 20 independent runs using different random initializations, examining consistency of dominant genera defining each topic and sample-level topic distributions (mean beta correlation = 0.883) (Supplemental Fig. S2). Topics were characterized by their dominant genera (highest beta probabilities) and named based on their microbial composition and established associations with vaginal health states.

#### Differential abundance analysis of topics

Topic-level differential abundance testing was performed using beta regression, which is specifically designed for continuous proportions bounded between 0 and 1. Beta regression directly models the gamma proportions (topic mixture coefficients) as response variables, appropriately handling the compositional nature of topic data without artificial conversion to count space. Raw gamma proportions from the LDA model were epsilon-transformed using the Smithson and Verkuilen (2006)^53^ method to handle boundary values: y’ = (y × (n − 1) + 0.5) / n, where n is the sample size. Beta regression models with logit link were fitted using the betareg package in R, with the formula: Topic ~ Race + Stage + Histology + Age. Coefficients from logit-link beta regression represent changes in log-odds of topic proportions and were exponentiated to obtain odds ratios (ORs) for interpretability. Age was analyzed as a continuous variable (per year), with per-decade ORs calculated as OR_decade = exp(β_age × 10) for more intuitive clinical interpretation. Statistical significance was determined using Benjamini–Hochberg false discovery rate (FDR) correction across all tests within each clinical variable (FDR < 0.05).

#### Species-level *Lactobacillus* validation

To complement topic-level findings, we performed targeted species-level analyses focusing on key *Lactobacillus* species known to be important for vaginal health. The phyloseq object was filtered to include only *Lactobacillus* species, and relative abundances were calculated by dividing species counts by total reads per sample. We examined both detection rates (presence/absence) and relative abundances when present for *L. crispatus*, *L. iners*, *L. gasseri*, and *L. jensenii* across demographic and clinical variables. Detection rate comparisons are reported as odds ratios with 95% confidence intervals, while abundance comparisons are reported as median fold-changes with corresponding confidence intervals.

#### Additional analyses

Detection rates between groups were compared using Fisher’s exact tests, while relative abundances (when detected) were compared using Wilcoxon rank-sum tests due to non-normal distributions. For detection rate analyses, effect sizes were calculated as odds ratios with 95% confidence intervals, while for abundance comparisons, effect sizes were reported as fold-changes between group medians. FDR correction was applied across all species tested within each comparison type (detection vs abundance). Statistical significance was set at FDR q < 0.05, with trends reported for FDR q < 0.10. Alpha and beta diversity metrics were computed using ASV-level count tables after quality filtering and taxonomic assignment. Phylogeny-based metrics (weighted UniFrac) were calculated using the ASV phylogenetic tree constructed during the DADA2 pipeline^36^. Alpha diversity (Shannon diversity index and Faith’s phylogenetic distance) was calculated using phyloseq^54^ (v1.48.0) and picante^55^ (v1.8.2) packages, while beta diversity was assessed using Jaccard, Bray-Curtis, UniFrac, and weighted UniFrac dissimilarity metrics calculated with the vegan package^56^ (v2.6.4), with principal coordinate analysis (PCoA) performed for visualization. Alpha diversity metrics were tested for normality using Shapiro–Wilk tests, and differences by demographic and clinical variables were assessed using appropriate parametric (ANOVA) or non-parametric (Kruskal–Wallis) tests. Differences in community composition between groups (beta diversity) were tested using permutational multivariate analysis of variance (PERMANOVA) implemented via the adonis2 function^57^ in the vegan R package^56^ with 999 permutations. PERMANOVA models were run separately for each demographic and clinical variable (race, age group, cancer stage, histology type) to assess individual effects on community structure, as simultaneous inclusion of all covariates was not feasible given sample size constraints and correlations among covariates (Supplemental Fig. S3). Effect sizes (R²) representing the proportion of variance explained by each variable were calculated and reported. *P*-values from separate PERMANOVA tests were adjusted for multiple comparisons using the false discovery rate (FDR) method.

#### Sensitivity analysis

To assess the robustness of observed racial differences to sample size imbalance, we conducted a downsampling sensitivity analysis. In 1000 iterations, NH-white participants were randomly subsampled to match the NH-Black sample size, and key race-associated microbiome features were re-evaluated. Distributions of effect estimates and *p*-values across iterations were examined to assess the stability of effect direction under balanced sampling (Supplemental Fig. S4). To further evaluate the robustness of key genus- and ASV-level findings to the high-zero structure of 16S count data, we conducted a focused zero-inflated sensitivity analysis for five pre-specified features: ASV54 (Actinomycetaceae), Actinotignum, Cutibacterium, Peptoniphilus, and Anaerococcus. For ASV54 (13% detection prevalence), a zero-inflated Gaussian model (fitZig, metagenomeSeq) was applied to CSS-normalized counts; for the four genus-level features (38–93% detection prevalence), MaAsLin2 with log-transformed relative abundance was used. A hurdle-style two-part decomposition separating presence/absence from conditional abundance was additionally applied to all five features. Results are presented in Supplemental Table S4.

#### Data visualization and statistical software

All statistical analyses were performed in R (v4.4.0) using the following packages: *phyloseq* (v1.48.0) for microbiome data handling^54^, *topicmodels* (v0.2.17*)* for LDA implementation^50^, *tidytext* (v0.4.3) for topic model interpretation^51^, rstatix for Fisher's exact and Wilcoxon rank-sum tests^58^, *betareg* (v3.2-4)^59^ for beta regression analysis, *ggplot2* (v3.5.2) for visualization^60^, and *cowplot* (v1.2.0) for multi-panel figures^61^. Statistical significance was set at FDR-adjusted q < 0.05 unless otherwise specified.

### Institutional review board

The study was conducted in accordance with the Declaration of Helsinki and approved by the Institutional Review Board (or Ethics Committee) of Duke University (Pro00101872).

### Informed consent

This study was approved by Duke University and the participating registries’ Institutional Review Boards (Pro00101872). All participants included provided informed consent.

## Results

### Cohort characteristics

A total of 132 OC patients were included in the analysis (Table 1); 83.3% NH-white (*n* = 110) and 16.7% NH-Black (*n* = 22). The median age was 61.5 years (range 30–79), with 83.3% of patients 50 years or older at diagnosis. Compared to NH-white patients, NH-Black patients were more likely to report lower annual household income (22.7% vs 7.3% reporting <$20,000), unemployment (13.6% vs 2.7%), or disability (27.3% vs 2.7%). About 72.7% of NH-Black patients were widowed, divorced, separated, or never married, compared to 32.7% of NH-white patients. Most patients were diagnosed with late-stage disease (FIGO stage III–IV; 68.9%), with a higher proportion of late-stage diagnosis among NH-Black compared with NH-white patients (77.3% vs 67.3%). Type II epithelial tumors were the most common histologic subtype (60.6%).

### Microbiome diversity

We evaluated vaginal microbiome diversity using both alpha (Shannon index) and beta (weighted UniFrac distance) diversity metrics (Supplemental Fig. S5, Supplemental Table S1). Alpha diversity showed no significant differences across race, age, stage, or histologic subtype (all *p* > 0.05, ANOVA; Supplemental Fig. S5A, C, E, G). Beta diversity analysis using weighted UniFrac distances and PERMANOVA revealed a significant association between histologic subtype and overall vaginal microbiome composition (R² = 0.018, *p* = 0.048; Supplemental Fig. S5H), with Type II epithelial tumors showing distinct community structure compared to Type I epithelial/Other tumors. In contrast, race (R² = 0.010, *p* = 0.22), age category (R² = 0.012, *p* = 0.14), and stage (R² = 0.005, *p* = 0.67) showed no significant associations with beta diversity (Supplemental Fig. S5B, D, F). Extraction batch showed no significant effect on community composition (R² = 0.004, *p* = 0.74), indicating good technical quality control. Tests for homogeneity of multivariate dispersion showed no significant differences across groups (all *p* > 0.05), validating the PERMANOVA results.

### Vaginal microbiome composition and relative abundance

**Fig. 2:**
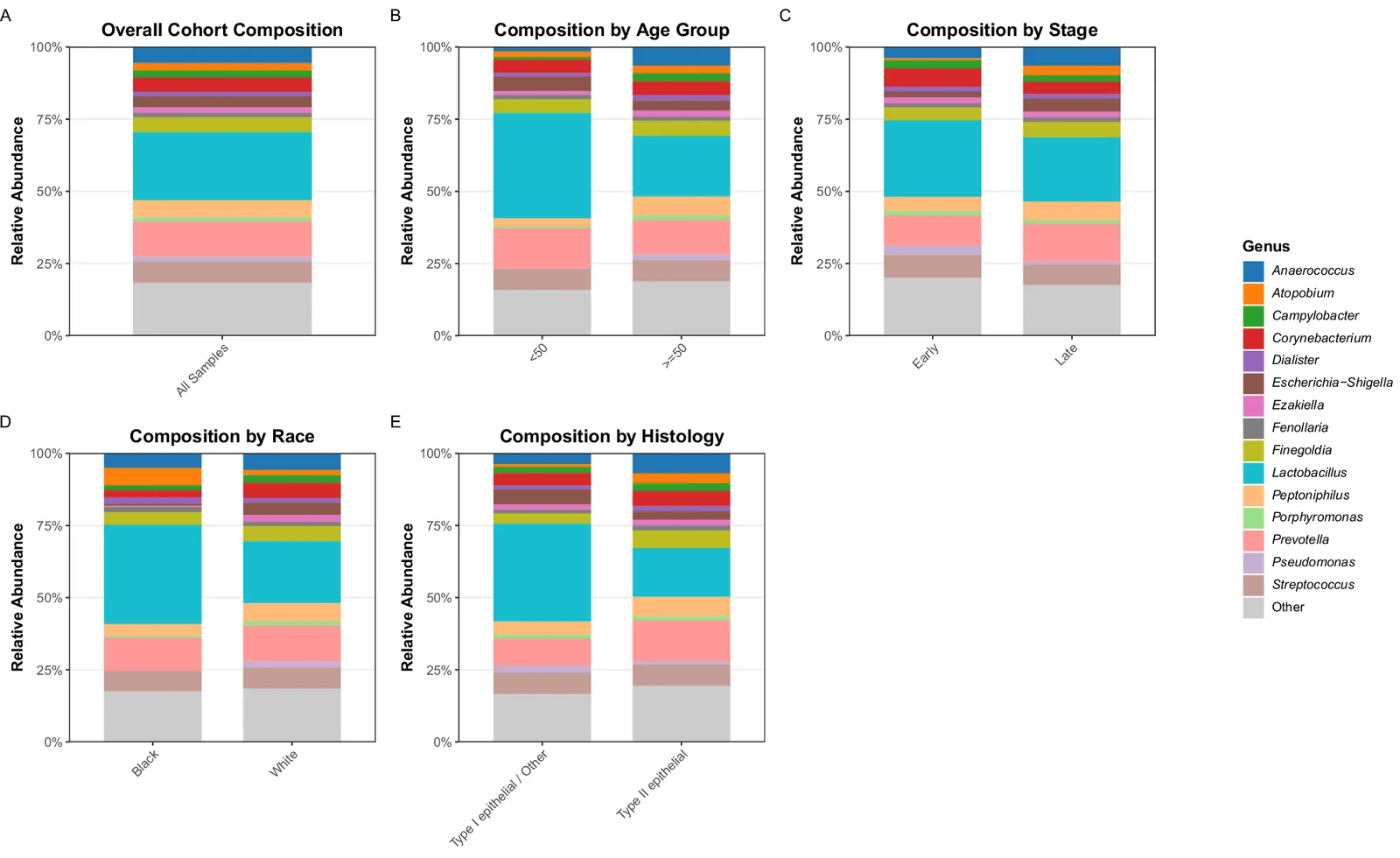
Vaginal microbiome composition across demographic and clinical variables. Stacked bar charts showing the relative abundance of major bacterial genera in OC patients. **A** Overall cohort composition, **B** composition by age group (<50 vs ≥50 years, **C** composition by cancer stage (Early vs Late), **D** composition by race (NH-Black vs NH-white), and **E** composition by histologic subtype (Type I epithelial + Other vs Type II epithelial).

Vaginal microbiome overall composition analysis at the genus level (Fig. 2, Supplementary Data 1) showed *Lactobacillus* as the most common genus by mean relative abundance (mean: 23.5%, median: 0% [IQR: 0–44.5%], prevalence: 47.7%), followed by *Prevotella* (mean: 12.3%, median: 4.9% [IQR: 0.6–23.6%], prevalence: 93.9%) and other anaerobic genera including *Peptoniphilus* (mean: 6.0%, prevalence: 95.5%), *Anaerococcus* (mean: 5.6%, prevalence: 90.9%), and *Finegoldia* (mean: 5.1%, prevalence: 96.2%). Supplemental Figure S6 displays genus-level microbiome profiles for individual participants, illustrating substantial inter-individual heterogeneity in dominant taxa. The difference between mean and median abundance for *Lactobacillus* (23.5% vs 0%) indicated a bimodal distribution (Supplemental Fig. S7), with this genus being either absent/minimal or highly dominant across individuals. This pattern reflects a cohort characterized by low *Lactobacillus* dominance, with *Lactobacillus* species present in less than half of patients, and widespread prevalence of anaerobic bacteria (>90% prevalence for most genera).

#### Age-related differences

Older patients ≥50 years had significantly higher prevalence of *Actinotignum* compared to younger patients (<50 years: prevalence 31.8%; ≥50 years: prevalence 68.2%, FDR q = 0.007), though mean relative abundances remained low in both groups (<50: mean 2.0%, median 0%; ≥50: mean 2.7%, median 0%). There were no other significant or trending associations by age group.

#### Cancer stage differences

Early-stage patients had significantly higher prevalence of *Cutibacterium* compared to late-stage patients (prevalence: 61.0% vs 33.0%, FDR q = 0.048). There were no other significant or trending associations by disease stage.

#### Racial differences

NH-Black patients had a 5-fold higher prevalence of a single Actinomycetaceae-classified amplicon sequence variant (ASV54) compared to NH-white patients (40.9% vs 8.2%, FDR q = 0.005), representing the most robust compositional difference observed in the cohort. NH-Black patients also had higher *Lactobacillus* prevalence (68.2% vs 43.6%) and mean abundance (mean 34.4% vs 21.4%), though this did not reach statistical significance (FDR q = 0.271). Notably, while overall *Lactobacillus* abundance appeared higher in NH-Black patients, this pattern was driven primarily by differences in *Lactobacillus* species composition (detailed in species-level analysis below). There were no other significant or trending racial differences in microbial composition.

#### Histologic subtype differences

There were no significant or trending associations between microbial composition and histologic subtype (all FDR q > 0.25).

### Topic modeling results

#### Identification of vaginal microbial topics

**Fig. 3:**
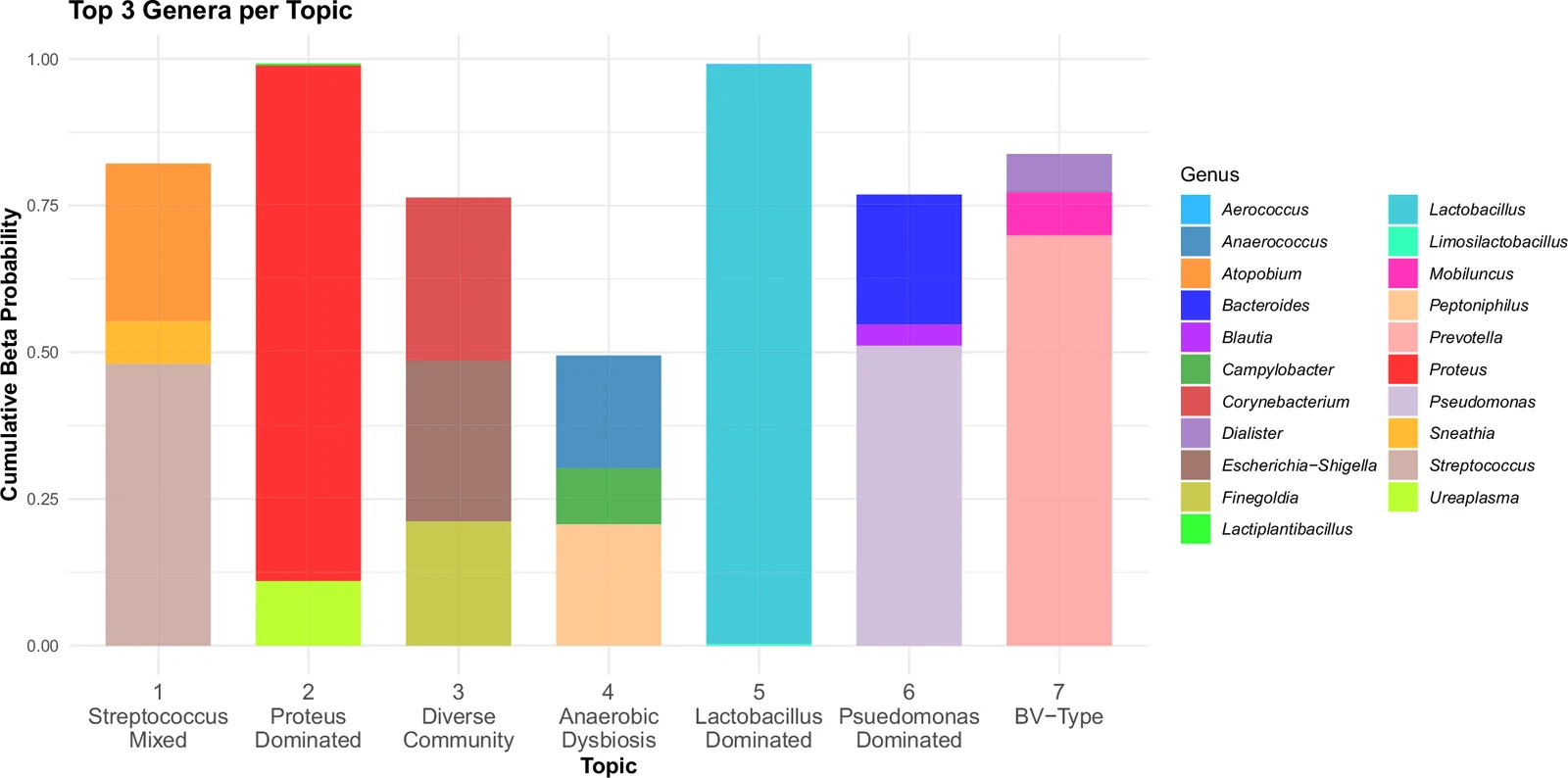
Vaginal microbiome topic signatures identified by latent Dirichlet allocation. Bar chart displaying the top 3 genera per microbial topic based on beta probability values. Seven distinct topics were identified: Topic 1 (*Streptococcus* Mixed)—moderate dysbiosis characterized by *Streptococcus* (48.1%), *Atopobium* (26.9%), and *Sneathia* (7.2%); Topic 2 (*Proteus*-dominated)—severe pathogenic dysbiosis dominated by *Proteus* (87.9%); Topic 3 (Diverse Community)—intermediate dysbiosis with *Corynebacterium* (27.8%), *Escherichia-Shigella* (27.5%), and *Finegoldia* (21.2%); Topic 4 (Anaerobic Dysbiosis)—anaerobic dysbiosis led by *Peptoniphilus* (20.7%) and *Anaerococcus* (19.3%); Topic 5 (*Lactobacillus*-dominated)—healthy signature nearly exclusively composed of *Lactobacillus* (98.6%); Topic 6 (*Pseudomonas*-dominated)—potentially representing opportunistic growth with *Pseudomonas* (51.2%); Topic 7 (BV-Type)—bacterial vaginosis-type dysbiosis dominated by *Prevotella* (70.0%).

LDA identified seven microbial topics using an elbow method approach, representing distinct co-occurring genus-level patterns that were biologically interpretable without excessive model complexity^62^ (Fig. 3, Supplemental Fig. S1). Topic 1 (*Streptococcus* Mixed) was characterized by moderate dysbiosis with *Streptococcus* (48.1%), *Atopobium* (26.9%), and *Sneathia* (7.2%), consistent with taxa commonly associated with bacterial vaginosis-like community structures^16,19^. Topic 2 (*Proteus*-dominated) was highly dominated by *Proteus* (87.9%) with minimal community diversity, reflecting a markedly dysbiotic profile, as *Proteus* species are associated with urogenital infections and opportunistic overgrowth^63^. Topic 3 (Diverse Community) showed intermediate dysbiosis led by *Corynebacterium* (27.8%), *Escherichia-Shigella* (27.5%), and *Finegoldia* (21.2%), representing heterogeneous, non-*Lactobacillus* communities characteristic of dysbiotic states^16^. Topic 4 (Anaerobic Dysbiosis) was dominated by *Peptoniphilus* (20.7%) and *Anaerococcus* (19.3%) with *Campylobacter* (9.5%), reflecting anaerobe-enriched community structures commonly observed in bacterial vaginosis and inflammatory conditions^64^. Topic 5 (*Lactobacillus*-Dominated) was nearly exclusively composed of *Lactobacillus* (98.6%), which can vary significantly in protective capacity depending on the specific species composition^16^. Topic 6 (*Pseudomonas*-dominated) was led by *Pseudomonas* (51.2%) with diverse anaerobes, consistent with opportunistic bacterial expansion in severely dysbiotic vaginal environments^65^. Topic 7 (BV-Type) was dominated by *Prevotella* (70.0%), consistent with classic bacterial vaginosis-type microbial profiles^16,19^. Collectively, these topics represent probabilistic patterns of microbial co-occurrence rather than mutually exclusive microbiome states. Topic 5 was the dominant community type in 26.8% of participants, while dysbiotic community types (Topics 1–4, 6, 7) dominated 73.2% of the cohort (Supplemental Fig. S8).

#### Topic-clinical variable associations

**Fig. 4:**
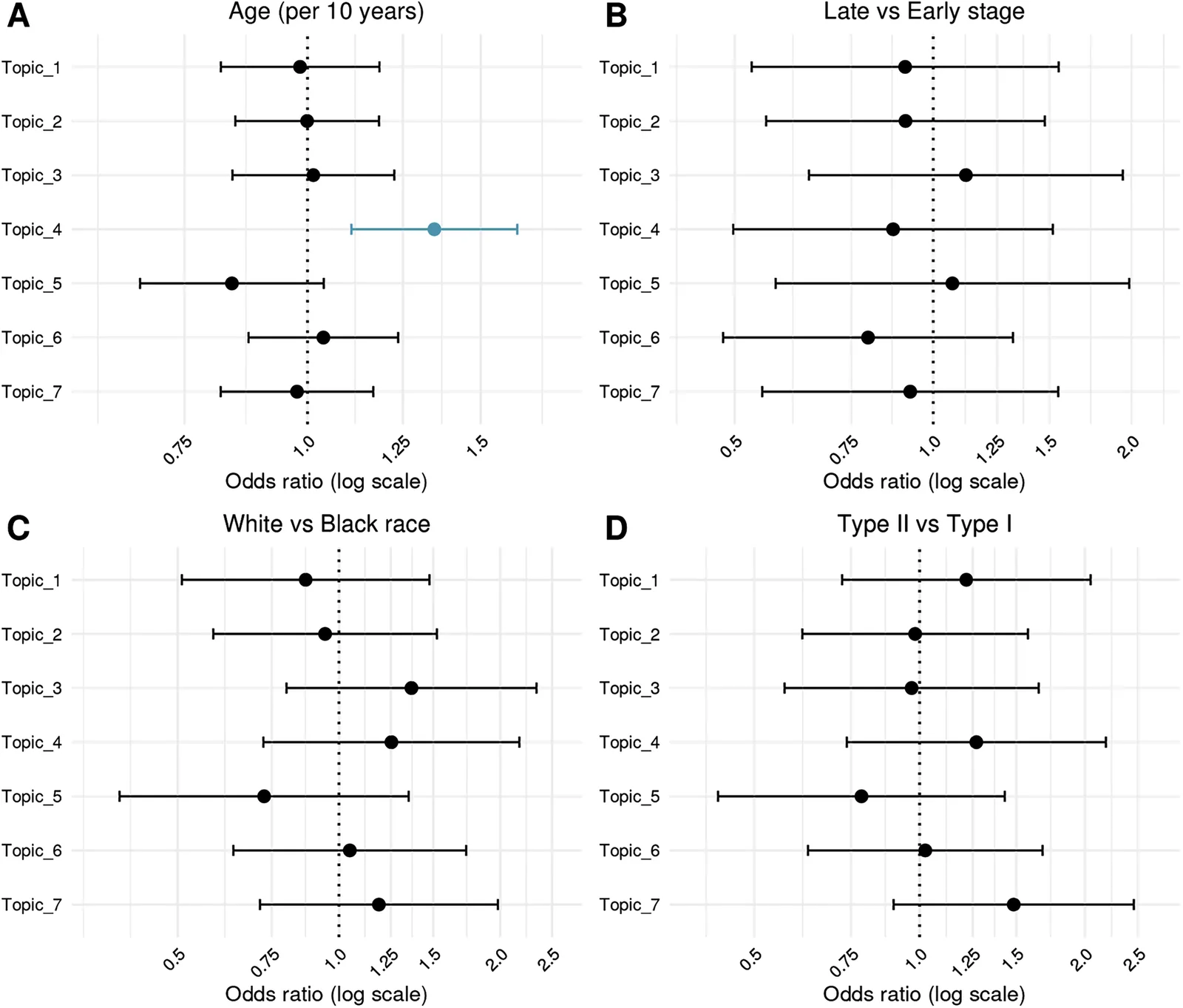
Topic-clinical variable associations using beta regression. Forest plots showing odds ratios (ORs) for microbial topics across clinical variables estimated using beta regression with a logit link. **A** Age (continuous, displayed as effect per 10 years for interpretability) showing Topic 4 (Anaerobic Dysbiosis) significantly enriched with increasing age (OR per decade = 1.35, 95% CI: 1.11–1.63, FDR q = 0.019; highlighted in blue). All other topics showed no significant age associations after FDR correction. **B** Disease stage comparison (Early stage I–II vs Late stage III–IV) showing no significant associations (all FDR q > 0.05). **C** Race comparison (Non-Hispanic white vs Non-Hispanic Black) showing no significant associations (all FDR q > 0.05). **D** Histologic subtype comparison (Type II epithelial vs Type I/non-epithelial) showing no significant associations (all FDR q > 0.05). Error bars represent 95% confidence intervals; statistical significance was determined using Benjamini–Hochberg FDR correction (q < 0.05).

Beta regression analysis of topic proportions revealed associations between microbial topic abundance and patient demographic and clinical characteristics (Fig. 4; Supplemental Table S2). Topic 4 (Anaerobic Dysbiosis, characterized by *Peptoniphilus* spp.) showed a significant positive association with age (β = 0.0297 per year, OR = 1.03 per year, 95% CI: 1.01–1.05, FDR q = 0.019). This corresponds to 34.6% higher odds of Topic 4 dominance per decade of life (OR = 1.35 per 10 years, 95% CI: 1.11–1.63), representing approximately 4-fold higher odds across the observed age range (31–79 years). No other topics showed statistically significant associations with age, race, cancer stage, or histologic subtype after FDR correction (all FDR q > 0.05).

### Species-level analysis of key *Lactobacillus* species

**Fig. 5:**
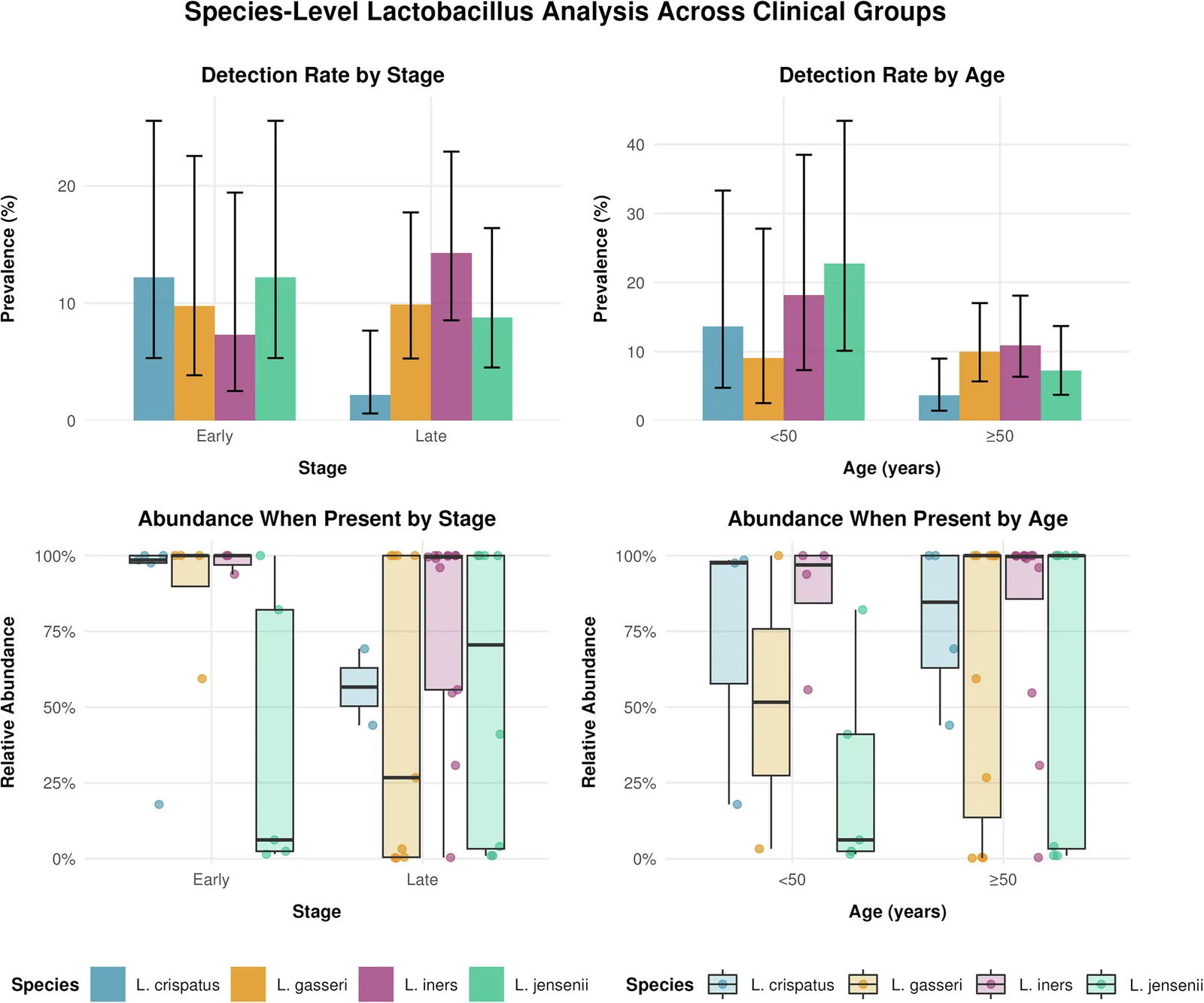
Species-level *Lactobacillus* validation of topic modeling findings. Detection prevalence (top panels) and relative abundance among detected samples (bottom panels) are shown for four predominant vaginal *Lactobacillus* species (*L. crispatus*, *L. iners*, *L. gasseri*, and *L. jensenii*) stratified by ovarian cancer stage (early vs late, left panels) and age category (<50 vs ≥50 years, right panels). Detection rates are presented as percentages with error bars representing 95% confidence intervals calculated using the Wilson score method. Sample sizes for each group: Early stage *n* = 41, Late stage *n* = 91, Age <50 *n* = 22, Age ≥50 *n* = 110. Boxplots summarize species-specific relative abundance among samples in which each species was detected, with individual observations overlaid as points. Statistical significance values are reported in Table 2. Statistical comparisons performed using two-sided Fisher’s exact tests (detection rates) and two-sided Wilcoxon rank-sum tests (abundance), with Benjamini–Hochberg FDR correction.

To complement the topic modeling findings, particularly regarding the *Lactobacillus*-dominated signature (Topic 5), we performed detailed species-level analyses of the four predominant vaginal *Lactobacillus* species (*L. crispatus*, *L. iners*, *L. gasseri*, *and L. jensenii*). Topic 5 abundance showed a strong positive correlation with species-level *Lactobacillus* abundance (Pearson r = 0.566, *p* < 0.001; Supplemental Fig. S9), validating the biological interpretability of our topic modeling approach. Given the bimodal distribution of *Lactobacillus* observed at the genus level, we examined both detection rates (presence/absence; Fig. 5A–C) and relative abundance when present for key *Lactobacillus* species (Fig. 5D–F). Corresponding estimates and 95% confidence intervals for key associations are summarized in Table 2 with all associations available in Supplemental Table S3.

**Table 2:** Species-level associations between vaginal *Lactobacillus* and clinical characteristics among ovarian cancer patients^a^

Species	Comparison	OR (95% CI)	Prevalence (%)	*p*-value	FDR q-value
*L. crispatus*	White vs Black	Undefined^b^ (0.28–∞)	6.4 vs 0.0	0.229	0.458
*L. crispatus*	Early vs late stage	6.25 (0.94–100.0)	12.2 vs 2.2	0.017	0.067
*L. crispatus*	Type I epithelial/Other vs Type II epithelial	4.17 (0.64–50.0)	9.6 vs 2.5	0.071	0.284
*L. crispatus*	<50 vs ≥50 years	4.17 (0.56–25.0)	13.6 vs 3.6	0.062	0.125
*L. jensenii*	<50 vs ≥50 years	3.70 (0.85–14.3)	22.7 vs 7.3	0.034	0.125
*L. iners*	Black vs white	2.63 (0.63–10.0)	22.7 vs 10.0	0.074	0.295
*L. iners*	Late vs early stage	2.08 (0.53–12.5)	14.3 vs 7.3	0.282	0.564
*L. iners*	<50 vs ≥50 years	1.82 (0.38–6.67)	18.2 vs 10.9	0.340	0.454
*L. gasseri*	Black vs white	1.56 (0.25–7.14)	13.6 vs 9.1	0.520	0.693

^a^Detection reflects the presence/absence of each species. *P*-values were calculated using Fisher’s exact tests, with false discovery rate (FDR) correction applied within species across comparisons. No associations met FDR q < 0.05; trends are reported for FDR q < 0.10. Odds ratios (ORs) with 95% confidence intervals were estimated using Fisher’s exact test. Prevalence values reflect detection rates for the first group listed relative to the second.

^b^OR not estimable due to zero detection events in the NH-Black group; lower bound of 95% CI = 0.28, upper bound not estimable.

#### Detection rates and clinical associations

Age-related patterns revealed differential effects on *Lactobacillus* species colonization. *L. crispatus* detection was higher in patients <50 years compared to those ≥50 years (13.6% vs 3.6%), although this difference did not reach statistical significance after FDR correction (FDR q = 0.125; Fig. 5A; Table 2). *L. iners* showed a similar but less pronounced age-related decline in detection (18.2% vs 10.9%; FDR q = 0.454). Detection patterns for *L. gasseri* and *L. jensenii* were comparable across age groups, with overlapping confidence intervals and no statistically significant differences. By disease stage, *L. crispatus* was detected more frequently in early-stage compared to late-stage patients (12.2% vs 2.2%), though this difference did not reach statistical significance after multiple testing correction (FDR q = 0.067; Fig. 5B). In contrast, *L. iners* showed higher detection in late-stage compared to early-stage disease (14.3% vs 7.3%), though this difference was not statistically significant (FDR q = 0.389). No significant stage-related differences were observed for *L. gasseri* or *L. jensenii*. Racial differences revealed opposing trends in *Lactobacillus* species distribution. *L. crispatus* was detected only among NH-white patients (6.4%) and not detected among NH-Black patients, while *L. iners* showed higher prevalence in NH-Black compared to NH-white patients (22.7% vs 10.0%). However, none of these racial differences reached statistical significance after FDR correction, and confidence intervals were wide (Fig. 5C; Table 2), reflecting limited power for species-level comparisons.

#### Abundance when present

Among samples in which *Lactobacillus* species were detected, relative abundance patterns differed by species. When present, *L. crispatus* consistently dominated the vaginal microbial community, with median relative abundance approaching 95–100% across demographic and clinical groups. *L. crispatus* abundance was significantly higher in early-stage compared to late-stage patients when detected (FDR q = 0.033; Fig. 5E), while age- and race-related differences in abundance were not statistically significant. In contrast, *L. iners* demonstrated greater variability in abundance when present, with median relative abundance ranging from approximately 55–95% across clinical groups and wider interquartile ranges (Fig. 5D–F). Abundance patterns for *L. gasseri* and *L. jensenii* were similarly variable and did not show consistent associations with demographic or tumor characteristics. These species-specific patterns are consistent with the recognized heterogeneity in functional and ecological roles among vaginal *Lactobacillus* species.

## Discussion

This comprehensive analysis of 132 OC patients identified widespread vaginal microbiome dysbiosis characterized by *Lactobacillus* depletion and enrichment of anaerobic bacterial taxa. We observed two key findings: significant age-related enrichment of anaerobic dysbiosis (Topic 4: *Peptoniphilus* and *Anaerococcus*), with approximately 35% higher odds per decade of life (OR = 1.35 per 10 years, 95% CI: 1.11–1.63, FDR q = 0.019), and marked racial differences in microbial composition, including a 5-fold higher prevalence of an Actinomycetaceae-classified amplicon sequence variant (ASV54) in NH-Black compared to NH-white patients (40.9% vs 8.2%, FDR q = 0.005). These findings demonstrate that vaginal microbiome composition varies substantially with age and race in post-treatment OC patients, with potential implications for understanding health disparities and clinical outcomes. This underscores the value of longitudinal studies to clarify temporal relationships and evaluate the potential for microbiome-informed interventions.

These findings align with and extend established literature on vaginal microbiome alterations in gynecologic malignancies. Previous OC studies by Jacobson et al. (2021) and Asangba et al. (2023) reported *Lactobacillus* depletion and anaerobic overgrowth, with only 24% of OC patients showing *Lactobacillus*-dominated microbiomes and significantly reduced abundance compared to controls (approximately 15% versus 30%)^28,66^. Our cohort showed similar patterns with 47.7% *Lactobacillus* detection and 23.5% mean relative abundance, falling substantially below rates typically observed in healthy reproductive-age women (70–80% dominance)^16^. Notably, *Gardnerella*, a genus frequently observed across multiple dysbiotic vaginal states and often implicated in anaerobic community restructuring^16,67^, was infrequently detected in this cohort and did not meet inclusion thresholds after filtering (Supplementary Data 1). This is consistent with prior work demonstrating substantial heterogeneity among non-*Lactobacillus*-dominated vaginal microbial profiles^16^. While histologic subtype reached statistical significance on PERMANOVA, all effect sizes were small (R² < 0.02), indicating that these variables explain a small fraction of overall microbiome variance, consistent with the high inter-individual variability characteristic of vaginal microbiome data and comparable to effect sizes reported in other population-based vaginal microbiome studies^68,69^.

Our analysis revealed significant age-related enrichment of anaerobic dysbiosis with Topic 4 (*Peptoniphilus* and *Anaerococcus*) showing 34.6% higher odds per decade of life (OR = 1.35 per 10 years, 95% CI: 1.11–1.63, FDR q = 0.019), a finding that has been limited in prior OC-focused microbiome studies. The compositional patterns we observed are consistent with prior work by Ravel et al., which established that healthy Black women are more likely to have non-*Lactobacillus*-dominated communities, with only 61.9% exhibiting *Lactobacillus*-dominated communities compared to 89.7% of white women^16^. However, no known studies have examined racial differences in vaginal microbiome composition among OC patients. We identified a single amplicon sequence variant (ASV54) classified within the Actinomycetaceae family that exhibited approximately 5-fold higher prevalence in NH-Black compared to NH-white patients (40.9% vs 8.2%, FDR q = 0.005), representing, to our knowledge, a novel race-associated microbial signature in the OC population. This finding remained robust in downsampling sensitivity analyses designed to account for sample size imbalance (Supplemental Fig. S4). Zero-inflated sensitivity analyses further confirmed the robustness of the ASV54 and age-associated findings across alternative modeling frameworks (Supplemental Table S4). Additionally, we observed that NH-Black patients had higher overall *Lactobacillus* prevalence (68.2% vs 43.6%) and mean abundance (34.4% vs 21.4%), though this difference reflected primarily *L. iners* rather than *L. crispatus*. While these species-level patterns provide important biological context about the *Lactobacillus* composition differences, larger studies will be needed to more fully characterize species-specific associations with demographic and clinical variables. The microbial topics identified here broadly correspond to established community state types (CSTs) described by Ravel et al., with Topic 5 mapping to *Lactobacillus*-dominated CSTs and Topics 1, 4, and 7 reflecting variants of the diverse anaerobic CST IV, consistent with prior work applying LDA-derived community groupings to examine microbiome-outcome relationships^70^.

The significant age-related enrichment of anaerobic dysbiosis (Topic 4: *Peptoniphilus* and *Anaerococcus*) reveals important biological pathways linking aging, microbiome composition, and cancer outcomes. These anaerobic bacteria are associated with bacterial vaginosis and chronic wound infections, where they act synergistically with other microbes to impair normal healing processes and contribute to chronic inflammatory states^65^. The magnitude of this age effect suggests that age-related hormonal changes, particularly declining estrogen in postmenopausal women^16^, drive community-level microbiome shifts toward anaerobic dysbiosis in OC patients. However, our use of chronological age as a proxy for menopausal status represents a limitation. Studies examining menopausal effects on the vaginal microbiome have documented significant declines in *Lactobacillus* prevalence and abundance with menopausal transition^71–73^, suggesting that some of our observed age-associated patterns may reflect hormonal status rather than chronological age per se. These findings are consistent with the oncobiome concept in which pathogen-driven disruptions create persistent microbial alterations that may promote chronic inflammation and carcinogenic processes^11^.

Our complementary species-level analyses revealed differential *Lactobacillus* composition patterns across demographic groups. While these associations did not reach statistical significance in our cohort, they provide valuable biological context given the well-established functional differences between *Lactobacillus* species. *L. crispatus* produces predominantly D-lactic acid, which has been associated with enhanced pathogen suppression compared to the L-lactate produced by *L. iners*^74,75^. *L. iners* dominance is associated with intermediate vaginal health states and increased susceptibility to dysbiotic transitions^76^. Recent mechanistic studies suggest that *L. iners* may actively promote treatment resistance through metabolic reprogramming^77^. Given these known biological distinctions, the observed compositional patterns—including higher *L. iners* prevalence in NH-Black patients and lower *L. crispatus* detection in older patients and those with advanced disease—merit follow-up investigation in larger cohorts with sufficient power to detect species-level associations.

The identification of an Actinomycetaceae-classified ASV (ASV54) showing 5-fold higher prevalence in NH-Black patients represents, to our knowledge, a novel microbial signature that may be associated with social and clinical factors affecting this population. Actinomycetaceae-classified taxa have been linked to pelvic inflammatory disease and chronic genital inflammation^78^, and recent work demonstrates that certain Actinobacteria species can metabolize estrogen and steroid hormones^79^. This highlights potential mechanisms through which this microbial signature could influence the local inflammatory or hormonal microenvironment in OC.

Taken together, our findings highlight a potentially important role for vaginal microbiome patterns in shaping OC prognosis, an area that warrants further study. Importantly, given that Black women experience later-stage diagnosis^80^, more aggressive tumor biology^81^, and reduced survival rates^14^, differential microbiome patterns may represent one of several biological pathways through which social, structural, and clinical inequities become biologically embedded. Efforts to more comprehensively characterize the vaginal microbiome and to identify targetable microbial pathways may provide valuable intervention opportunities. While there is currently insufficient evidence to support routine probiotic recommendations for OC patients^82^, additional studies building on our findings could inform species-specific, context-dependent strategies, rather than generic interventions, to modulate the vaginal microbiome in ways that may ultimately improve outcomes.

Several important limitations should be considered. The cross-sectional design prevents the determination of causality or temporal relationships between microbiome changes and disease characteristics. Accordingly, we cannot infer whether observed microbiome features preceded cancer development, arose as a consequence of malignancy or treatment, or reflect aging-related shifts independent of cancer. While the majority of patients in the cohort had received treatment at the time of sample collection, treatment timing relative to microbiome sampling varied, limiting our ability to disentangle treatment-related effects from disease-associated or host-related influences. Similarly, we cannot fully disentangle age-related effects from hormonal changes associated with menopause in this cross-sectional study, as we used age as our primary aging variable.

Additional limitations relate to biospecimen collection and timing. Vaginal samples were self-collected and returned by mail, resulting in unavoidable variability in collection timing, shipment duration, and processing schedules that could not be fully standardized. All samples were collected using stabilization media validated for ambient-temperature storage and were processed within manufacturer-recommended timeframes; however, some degree of pre-analytic variability is inherent to participant-driven, mail-based sampling. In addition, samples were collected at heterogeneous time points relative to ovarian cancer diagnosis and treatment, and complete diagnosis dates were not uniformly available across participating state cancer registries, limiting our ability to precisely model time since diagnosis or treatment. These factors may contribute to unexplained variability in microbiome composition and limit causal inference, warranting cautious interpretation.

Taxonomy was assigned using Silva v138.1; we recognize that vaginal-species resolution can be limited with general-purpose databases, and therefore, we limited species-level inference to targeted *Lactobacillus* analyses. The sample size was relatively small for detecting subtle associations, particularly for NH-Black patients (*n* = 22), which limited statistical power for subgroup analyses. Although genus-level differential abundance analyses did not employ a zero-inflated framework as a primary approach, a focused sensitivity analysis confirmed consistent effect directions across zero-inflated and hurdle-style models for the five key findings reported here; the Cutibacterium stage-associated finding attenuated under zero-inflated modeling and should be interpreted as exploratory. Our 16S rRNA sequencing approach provides taxonomic composition but limited functional insight into microbiome activity. Methodological considerations, such as the exclusion of patients with recent antibiotic use, and focusing on patients at least 9 months post-diagnosis, potentially resulted in selecting for more stable microbiome profiles, and missing acute changes associated with initial diagnosis and treatment. The inclusion of patients across different treatment stages likely introduced clinical heterogeneity that could have attenuated some associations. An additional limitation is the potential for site- or registry-level confounding of observed racial differences. NH-Black patients in this cohort were geographically concentrated in the South (50.0%) and Northeast (40.9%), while NH-white patients were more evenly distributed across regions. Because registry catchment areas may reflect distinct environmental exposures, healthcare systems, and demographic compositions, geographic clustering cannot be fully disentangled from race in this sample.

Nevertheless, our study has several notable strengths that support the validity of our findings. The population-based recruitment from multiple state cancer registries enhances generalizability, while the multi-scale analytical approach combining traditional composition analysis, topic modeling, and species-level validation provided convergent evidence for our key findings. The self-collection methodology improved feasibility and reduced selection bias, and our rigorous exclusion criteria for antibiotic and suppository use minimized confounding factors. Together, these strengths support the internal validity of the observed associations while underscoring the need for longitudinal and mechanistic studies to further clarify causal relationships.

## Conclusions

We identified consistent vaginal microbiome dysbiosis among OC patients with notable age- and race-associated patterns that may have implications for disease progression and outcomes. These findings establish a strong foundation for future research investigating specific vaginal microbiome patterns beyond the species level in understanding and addressing OC progression and outcome disparities. If validated in larger, longitudinal studies, vaginal microbiome profiles could potentially serve as biomarkers for disease monitoring or therapeutic targets to improve outcomes, particularly for older patients and NH-Black patients who demonstrate the most pronounced dysbiotic signatures. While integration of microbiome assessment into clinical care is not yet warranted, continued investigation of species-specific and functionally relevant microbial patterns may ultimately inform more personalized approaches to OC management.

## Supplementary information

Supplementary Information

Description of Additional Supplementary files

Supplementary Data 1

## Data Availability

Raw 16S rRNA sequencing data generated in this study have been deposited in the NCBI Sequence Read Archive (SRA) under BioProject accession PRJNA1414650 (BioSamples SAMN60171617–SAMN60171748). Data are publicly available. Source data for all figures are provided in the Supplementary Data file. De-identified sample metadata and data dictionary are available at https://github.com/adeveaux/orchid-microbiome-analysis. Additional data are available from the corresponding author upon reasonable request.

## Code availability

Code for topic modeling (LDA) and differential abundance analysis (beta regression) is publicly available at https://github.com/adeveaux/orchid-microbiome-analysis.
