# Supplementary material for: Characterizing the composition and diversity of the vaginal microbiome in ovarian cancer
Source: Commun Med (Lond). 2026 Jul 10;6:573. doi: 10.1038/s43856-026-01771-8 (PMC13728130; doi:10.1038/s43856-026-01771-8)
Supplement: Supplementary file 1 [file 43856_2026_1771_MOESM1_ESM.pdf]

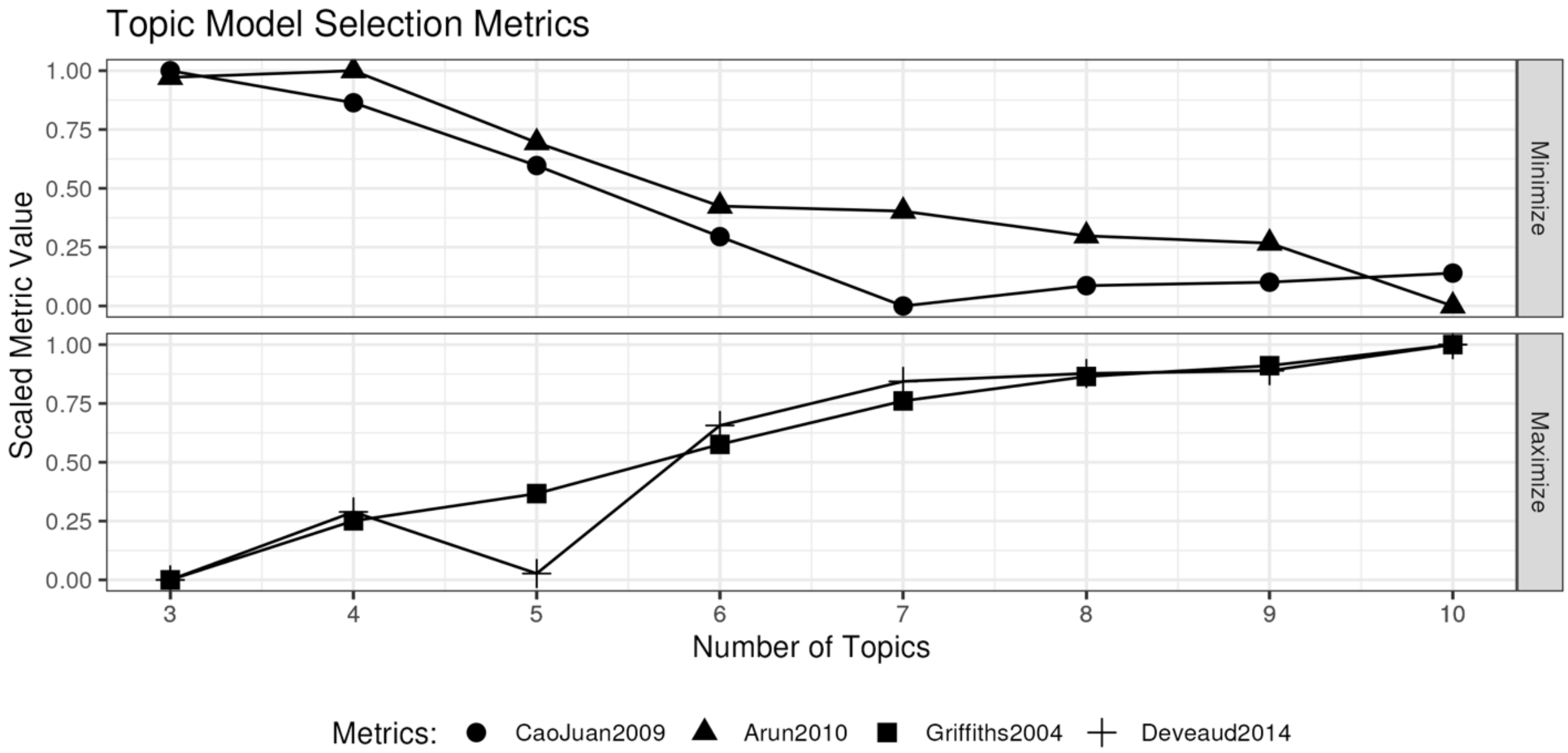

Supplemental Figure S1. Optimal topic number determination for vaginal microbiome LDA model. Evaluation metrics from Latent Dirichlet Allocation (LDA) topic modeling are plotted to identify the optimal number of latent topics representing the vaginal microbiome community structure. Four complementary metrics are shown: CaoJuan2009 and Arun2010 (minimized values indicate better model fit, top panel), and Griffiths2004 and Deveaud2014 (maximized values indicate better model fit, bottom panel) across a range of topic counts from 3 to 10 topics. Convergence of multiple optimization metrics is observed at 7 topics, indicating that this number provides the best balance between model complexity and biological interpretability. Based on this multi-metric analysis, 7 latent topics were selected for the final LDA model. Each topic corresponds to a distinct co-occurring taxa pattern in the vaginal microbiome.

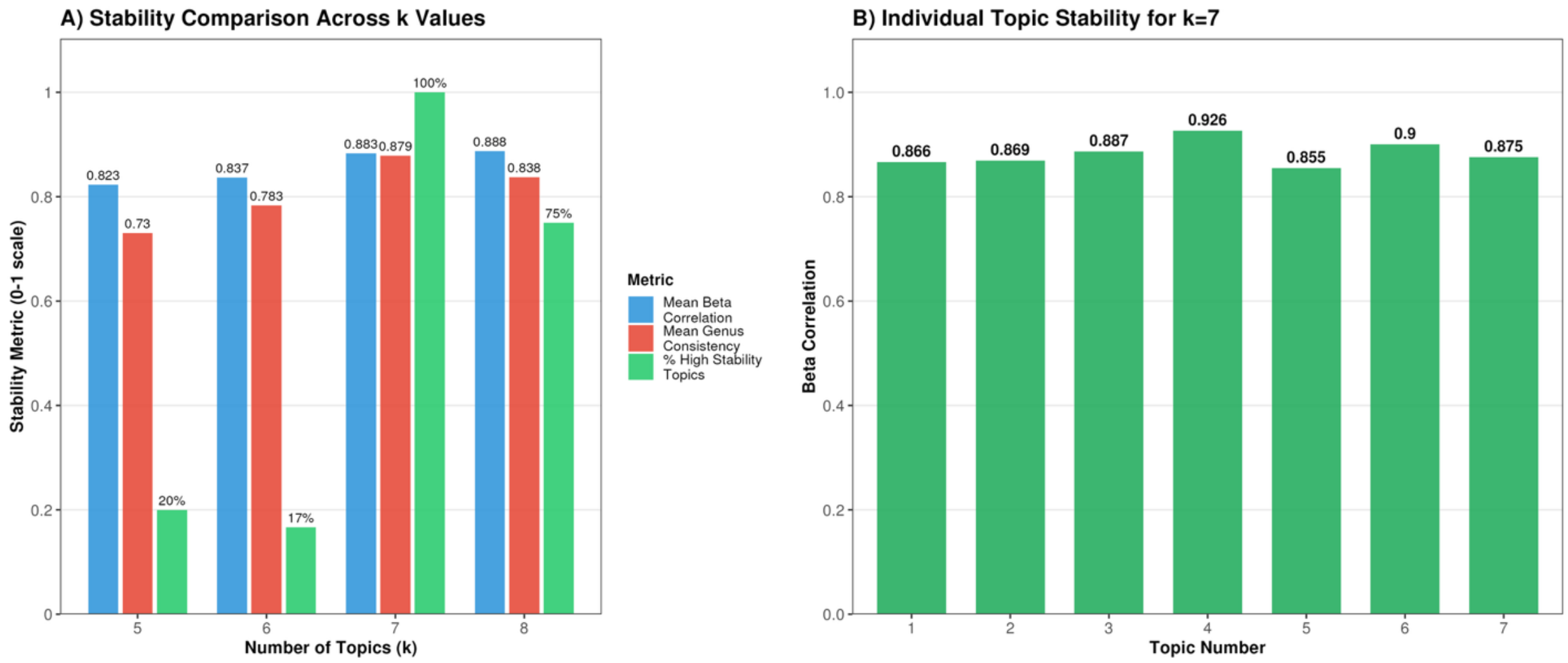

Supplemental Figure S2. Validation of Optimal Topic Number Through Stability Analysis. Stability analysis across k=5-8 topics using 20 independent LDA models with different random seeds. (**A**) Comparison of stability metrics across topic numbers. k=7 showed superior performance with mean beta correlation of 0.883 and mean genus consistency of 0.879. k=8 showed declining genus consistency (0.838) and reduced topic stability (75% vs 100%), confirming k=7 as optimal. (**B**) Individual topic stability for the selected k=7 model. All seven topics achieved high stability (correlation ≥0.85), indicating robust biological communities. (**C**) Dominant genus consistency showing that 6 of 7 topics (86%) maintained the same dominant genus in ≥80% of independent runs. Beta correlation measures similarity of genus probability distributions after optimal topic alignment. Genus consistency measures the frequency with which the same genus dominates each biological community across runs.

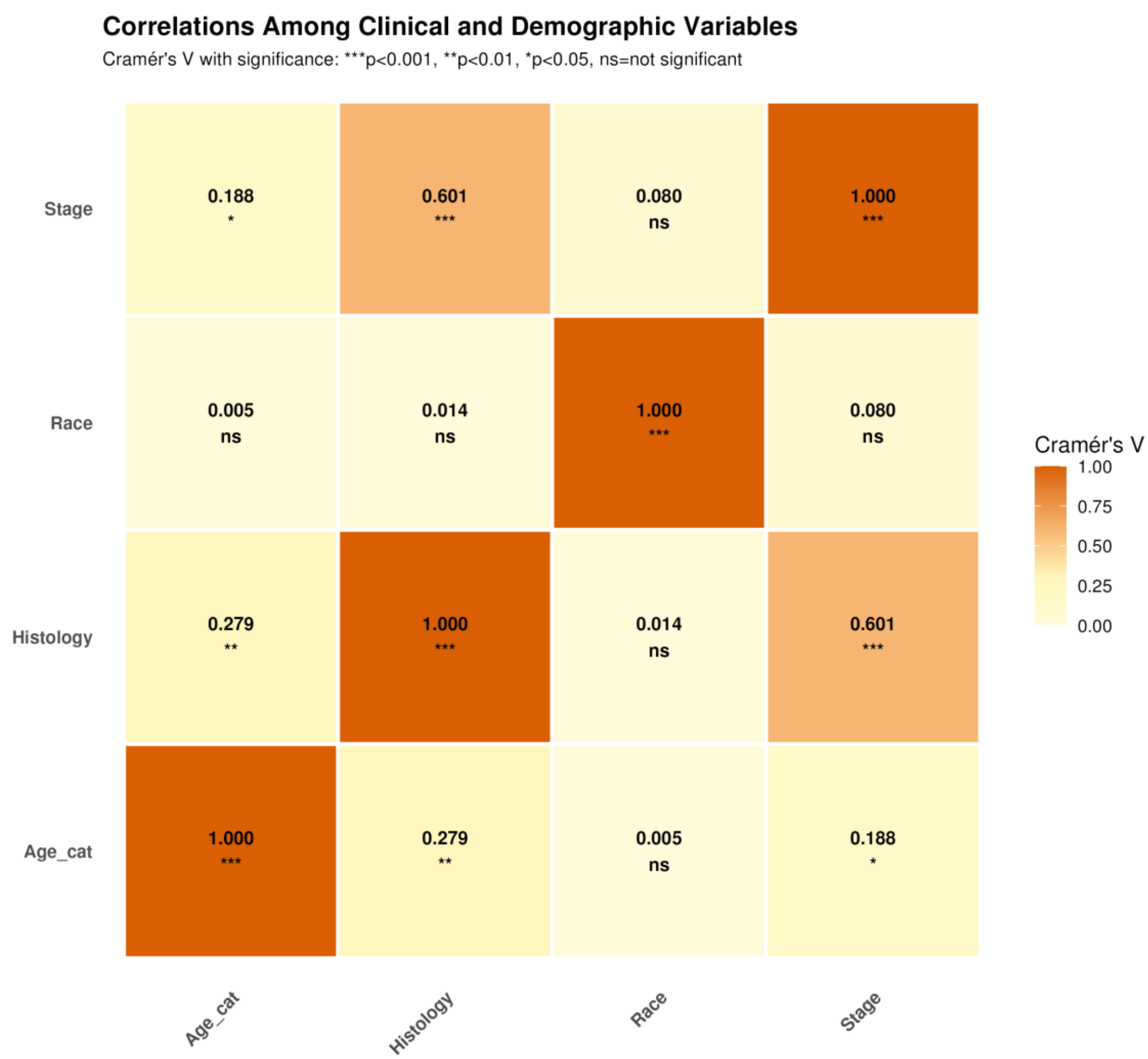

Supplemental Figure S3. Correlations among clinical and demographic variables. Heatmap displaying Cramér's V coefficients measuring associations between categorical variables, with statistical significance indicated by asterisks (***p<0.001, **p<0.01, *p<0.05, ns=not significant). Color intensity represents correlation strength (white=no correlation, dark orange=perfect correlation). Strong correlations were observed between cancer stage and histologic subtype (V=0.601, p<0.001), with moderate correlations between age category and histology (V=0.279, p=0.004) and between age category and stage (V=0.188, p=0.012). Race showed minimal correlation with all clinical variables (all V<0.08, p>0.4), supporting independent assessment of race-associated microbiome patterns.

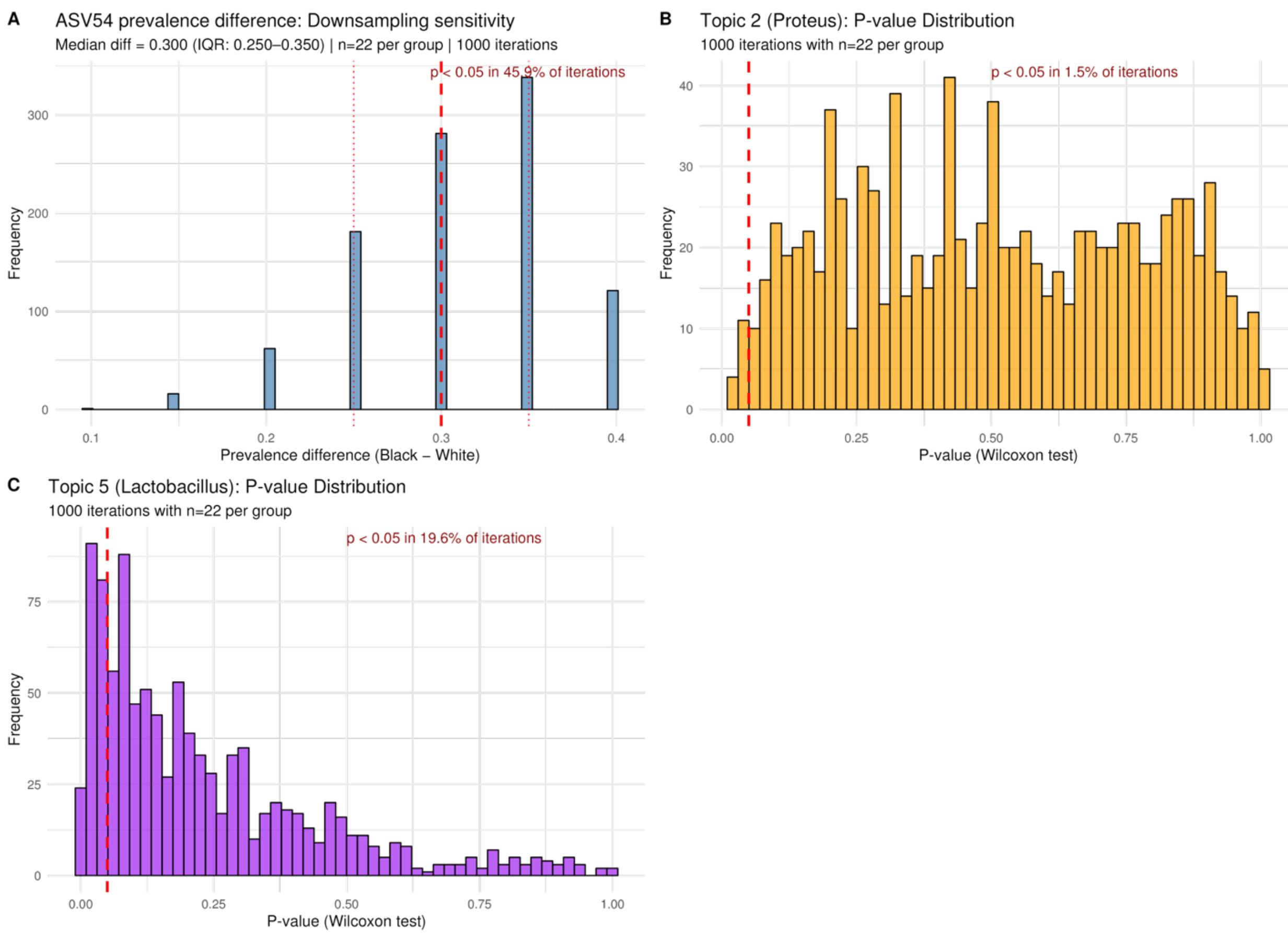

Supplemental Figure S4. Downsampling sensitivity analysis for racial comparisons. To evaluate the robustness of observed racial differences to sample size imbalance, we performed 1,000 iterations of downsampling, randomly selecting NH-White patients to match the NH-Black sample size in each iteration (n = 22 per group). **(A)** Distribution of prevalence differences for ASV54 (Actinomycetaceae-assigned ASV), calculated as Black minus White prevalence across iterations. Red dashed line indicates the median prevalence difference; red dotted lines indicate the interquartile range. Statistical significance ($p < 0.05$) was observed in 45.9% of iterations. **(B)** Distribution of p-values for Topic 2 (Proteus-dominated) associations with race across iterations. **(C)** Distribution of p-values for Topic 5 (Lactobacillus-dominated) associations with race across iterations. Red dashed vertical lines in panels B and C indicate the p = 0.05 threshold. Overall, results demonstrate consistent effect direction across downsampling iterations with reduced statistical power under balanced sample sizes.

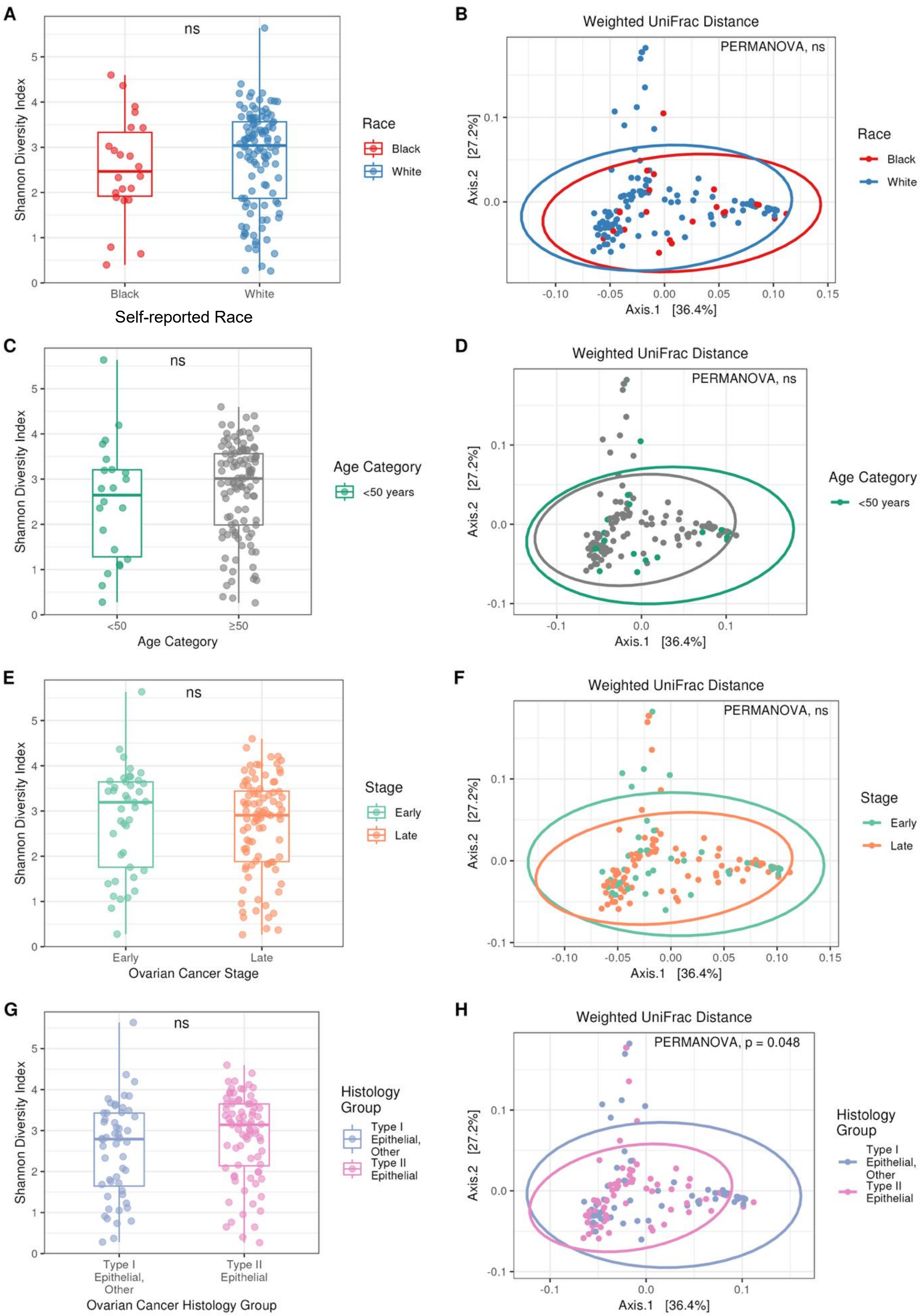

A
ns
Shannon Diversity Index
Race
Black
White
Self-reported Race
B
Weighted UniFrac Distance
PERMANOVA, ns
Axis.2 [27.2%]
Axis.1 [36.4%]
Race
Black
White
C
ns
Shannon Diversity Index
Age Category
<50 years
<50
≥50
Age Category
D
Weighted UniFrac Distance
PERMANOVA, ns
Axis.2 [27.2%]
Axis.1 [36.4%]
Age Category
<50 years
E
ns
Shannon Diversity Index
Stage
Early
Late
Ovarian Cancer Stage
F
Weighted UniFrac Distance
PERMANOVA, ns
Axis.2 [27.2%]
Axis.1 [36.4%]
Stage
Early
Late
G
ns
Shannon Diversity Index
Histology Group
Type I Epithelial, Other
Type II Epithelial
Ovarian Cancer Histology Group
H
Weighted UniFrac Distance
PERMANOVA, p = 0.048
Axis.2 [27.2%]
Axis.1 [36.4%]
Histology Group
Type I Epithelial, Other
Type II Epithelial

Supplemental Figure S5. Alpha and beta diversity analysis of vaginal microbiome across demographic and clinical variables. Shannon diversity index (left panels) and principal coordinate analysis based on weighted UniFrac distances (right panels) stratified by demographic and clinical variables. (**A-B**) Race (NH-Black vs NH-White; n=22 vs n=110), (**C-D**) Age category (<50 vs ≥50 years; n=41 vs n=91), (**E-F**) Cancer stage (Early vs Late; n=41 vs n=91), and (**G-H**) Histologic subtype (Type I epithelial/Other vs Type II epithelial; n=52 vs n=80). Ellipses in beta diversity plots represent 95% confidence intervals. PERMANOVA analysis revealed a significant association between histologic subtype and overall community composition (p=0.048; Panel **H**), while no significant associations were observed for race, age, or stage (all p>0.13; see **Supplementary Table S1**). Alpha diversity showed no significant differences across any demographic or clinical variable (all p>0.05, ANOVA). Tests for homogeneity of multivariate dispersion showed no significant differences in dispersion across groups (all p>0.05), validating the PERMANOVA approach.

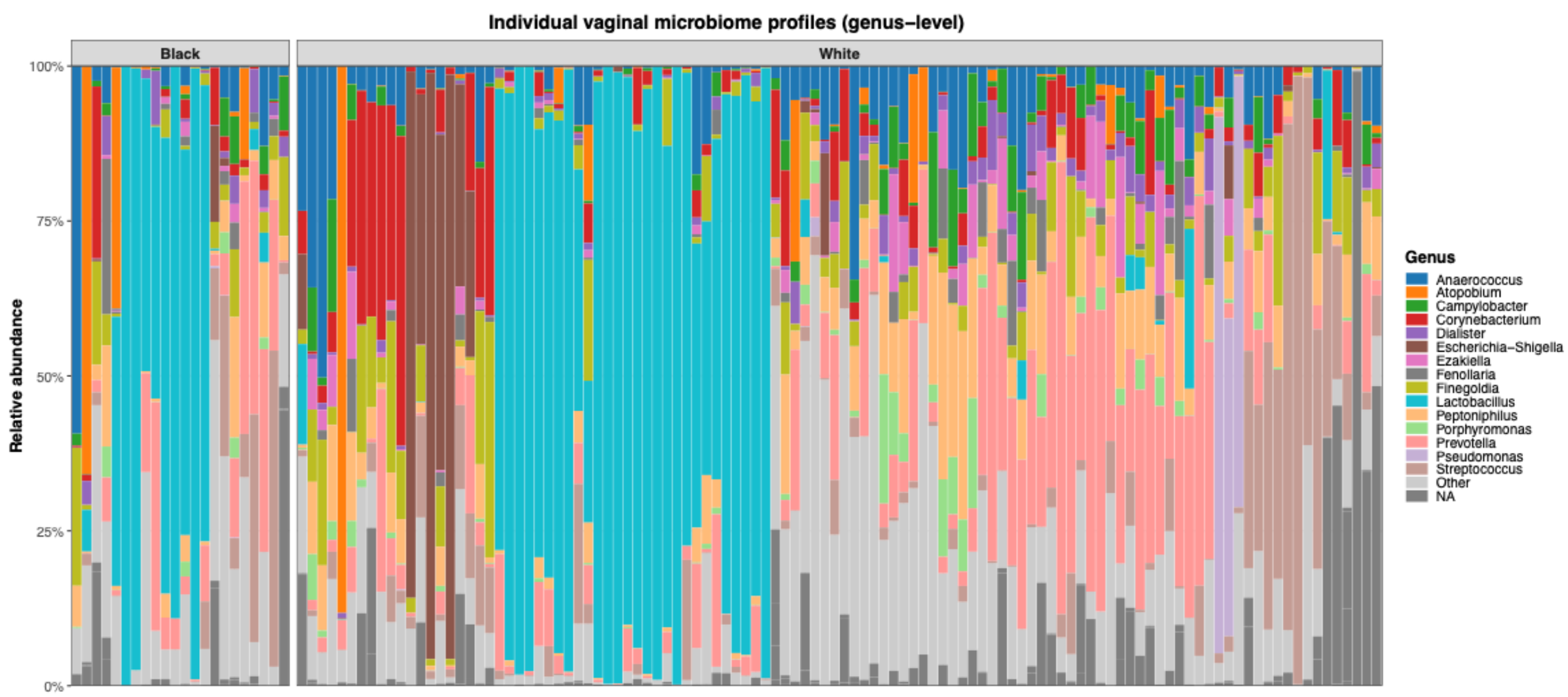

Supplemental Figure S6. Individual vaginal microbiome profiles at the genus level. Stacked bar plots show the relative abundance of vaginal bacterial genera for each individual participant, stratified by race (Black vs White). Each vertical bar represents one participant, and bars sum to 100% within individuals. Genera are shown at the genus level and ordered within race strata by dominant genus for visual clarity. Only the top 20 genera by mean relative abundance across the cohort are displayed; all remaining genera, including unclassified taxa, are collapsed into the “Other” category (grey). Colors are consistent with those used in main and supplementary microbiome composition figures. This visualization highlights substantial inter-individual heterogeneity in vaginal microbial community composition within and across racial groups.

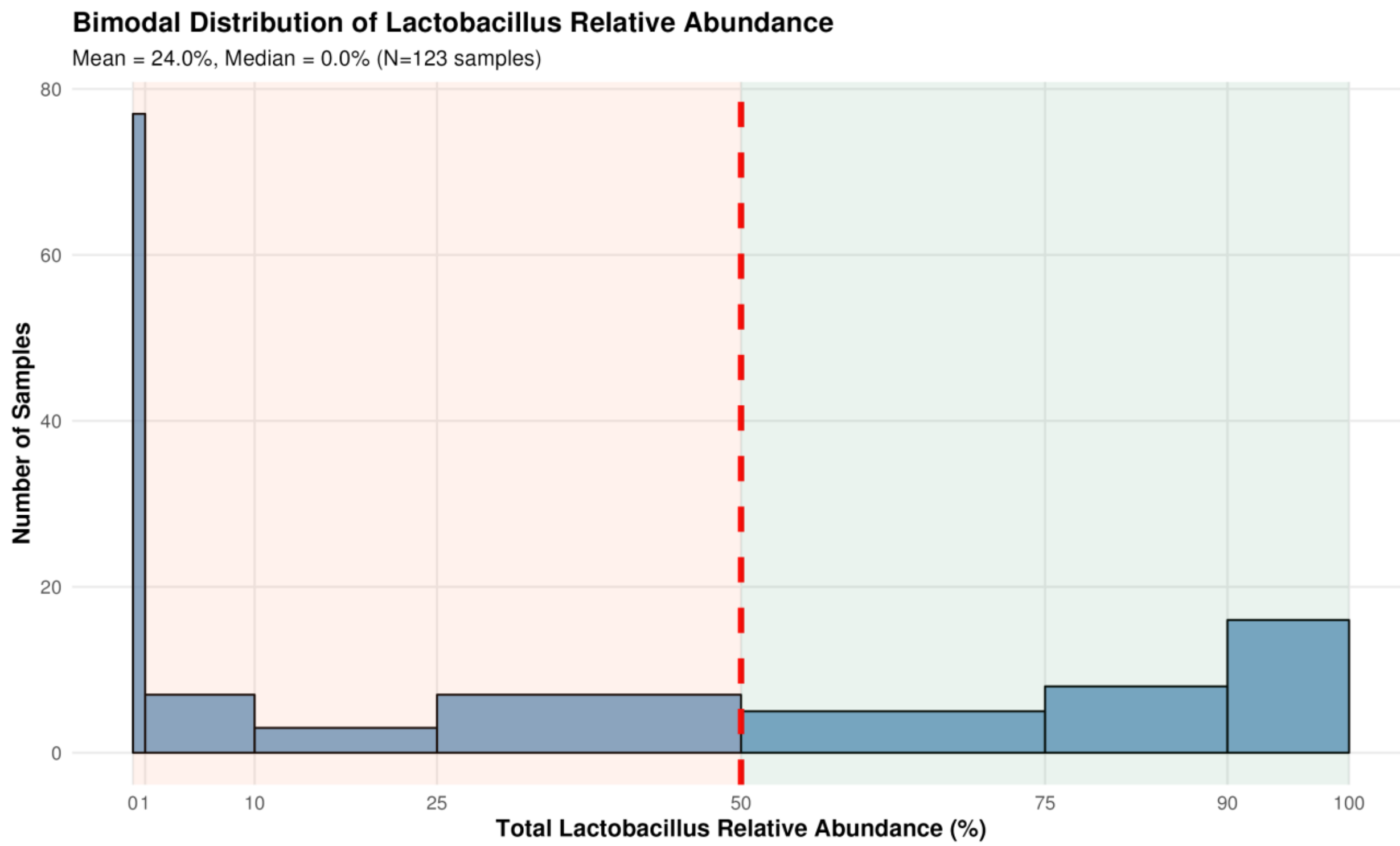

Supplemental Figure S7. Bimodal distribution of *Lactobacillus* relative abundance across the cohort. Histogram showing total *Lactobacillus* relative abundance per sample (N=123) with custom bin widths to highlight distributional patterns. The red dashed line indicates the 50% dysbiosis threshold. Two distinct peaks are evident: a left peak at <1% abundance containing 77 samples (62.6% of cohort, coral shaded region) representing *Lactobacillus*-depleted dysbiotic profiles, and a right peak at 90-100% abundance containing 16 samples (13% of cohort, green shaded region) representing *Lactobacillus*-dominated eubiotic profiles. The sparse middle region (1-90%) contains only 30 samples (24.4%), indicating that most participants exhibit one of two discrete community states rather than a continuum of intermediate profiles.

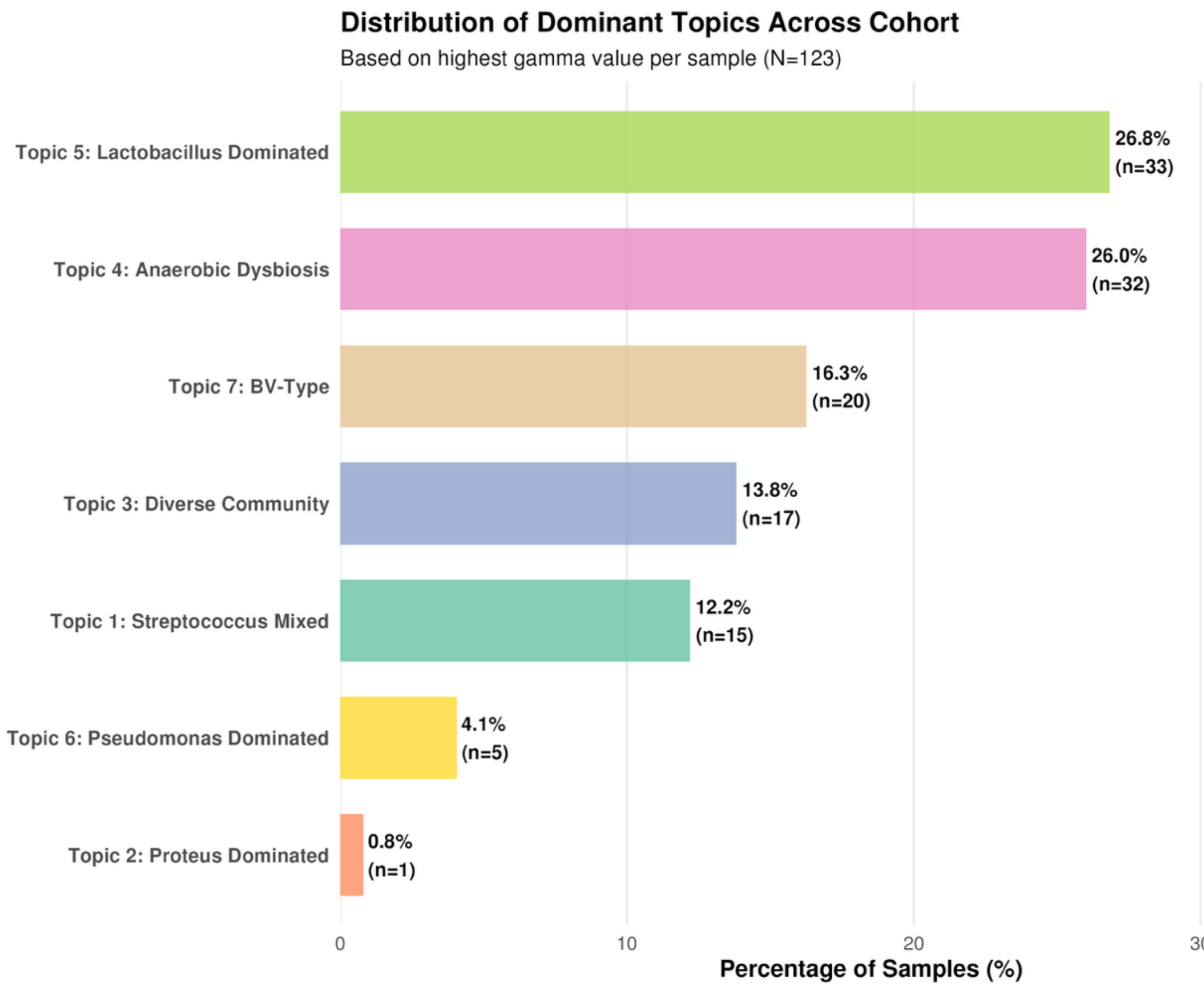

Supplemental Figure S8. Distribution of dominant microbial community topics across the cohort. Horizontal bar chart showing the percentage of samples (N=123) where each of the seven identified topics represents the dominant community type (highest per-sample gamma value). Topic 5 (*Lactobacillus* Dominated) was dominant in 26.8% of participants (n=33), representing the eubiotic community state. Dysbiotic community types dominated 73.2% of participants, with anaerobe-enriched communities (Topic 4: Anaerobic Dysbiosis, 26.0%, n=32; Topic 7: BV-Type, 16.3%, n=20) being most prevalent, followed by diverse polymicrobial communities (Topics 1, 3, 6, 2). This distribution demonstrates distinct community structure patterns consistent with the bimodal *Lactobacillus* abundance distribution (Supplementary Figure S7).

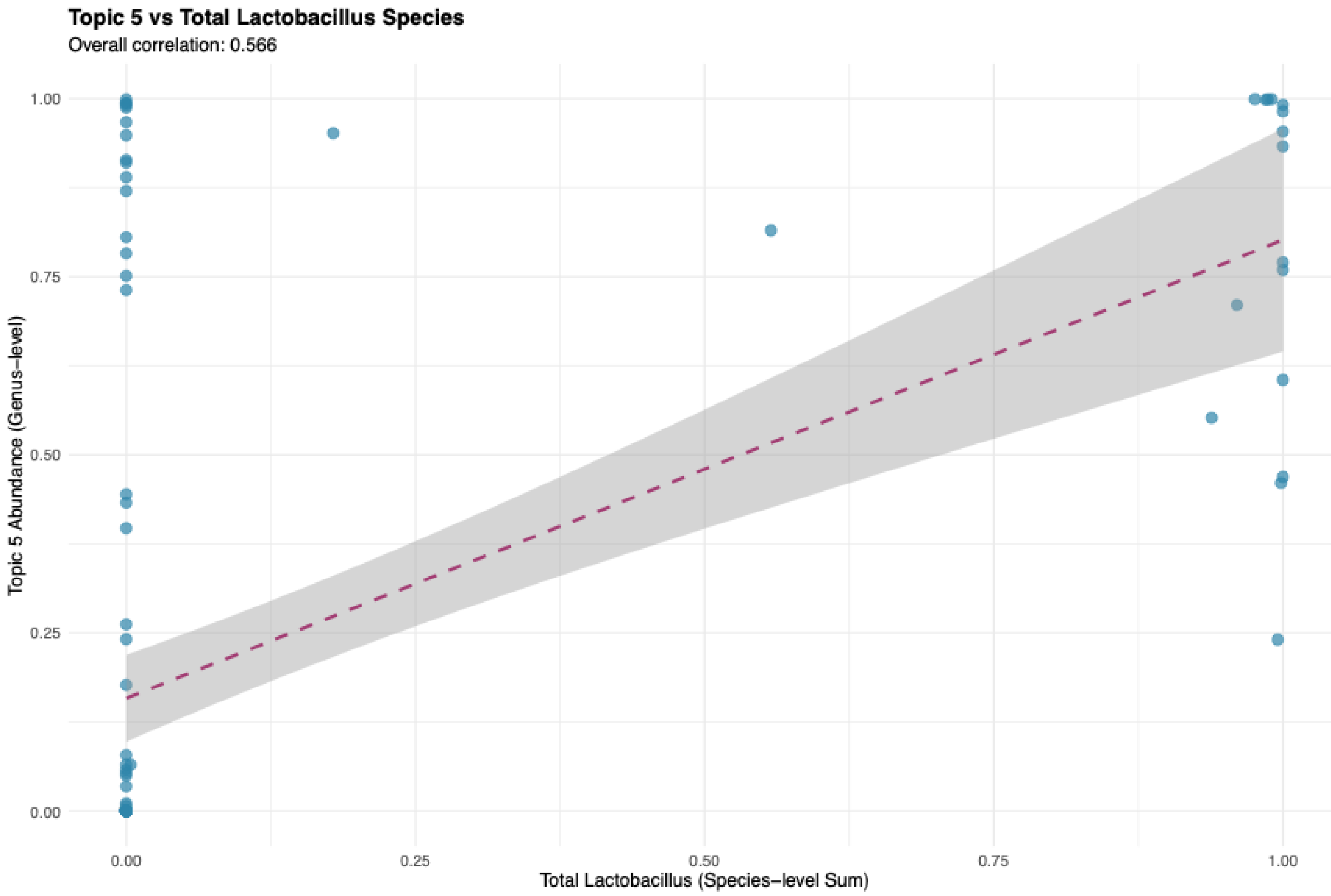

Supplemental Figure S9. Correlation between topic modeling (Topic 5) and species-level *Lactobacillus* abundance. Scatter plot showing the relationship between Topic 5 abundance (derived from genus-level topic modeling, y-axis) and total *Lactobacillus* abundance calculated from species-level analysis (x-axis). Each point represents one participant sample (N=132). The dashed red line represents the linear regression fit with 95% confidence interval (shaded gray region). Strong positive correlation (Pearson r=0.566, p<0.001) validates that Topic 5 accurately captures *Lactobacillus*-dominated community states, supporting the biological interpretability of the topic modeling approach. Topic 5 abundance and species-level *Lactobacillus* measurements are shown as proportions (0-1 scale).

Supplemental Table S1. PERMANOVA

Variable	Df	SumOfSqs	R2	F	Pr(>F)
Race (NH-Black vs NH-White)	1	0.01048	0.0103	1.37	0.217
Age (<50 vs ≥50 years)	1	0.01239	0.0122	1.62	0.137
Stage (Early vs Late)	1	0.00472	0.0046	0.62	0.673
Histology (Type I/Other vs Type II)	1	0.01828	0.018	2.38	0.048
Extraction Batch	1	0.00427	0.0042	0.56	0.741
Residual	126	0.96583	0.9506	NA	NA
Total	131	1.01598	1	NA	NA

Supplemental Table S2. Complete Beta Regression Results

Variable	Comparison	Topic	β	SE	OR	95% CI	P value	FDR
Age	Per year increase	Topic 1	-0.0017	0.0095	0.998	0.98-1.017	0.855	0.993
Age	Per year increase	Topic 2	-0.0001	0.0086	1	0.983-1.017	0.993	0.993
Age	Per year increase	Topic 3	0.0014	0.0097	1.001	0.983-1.021	0.887	0.993
Age	Per year increase	Topic 4	0.0297	0.0099	1.03	1.01-1.05	0.003	0.019
Age	Per year increase	Topic 5	-0.0177	0.011	0.982	0.962-1.004	0.107	0.374
Age	Per year increase	Topic 6	0.0037	0.0089	1.004	0.986-1.022	0.676	0.993
Age	Per year increase	Topic 7	-0.0025	0.0091	0.998	0.98-1.016	0.788	0.993
Race	White vs Black	Topic 1	-0.1431	0.2714	0.867	0.509-1.475	0.598	0.837
Race	White vs Black	Topic 2	-0.0596	0.2452	0.942	0.583-1.524	0.808	0.855
Race	White vs Black	Topic 3	0.3116	0.2741	1.366	0.798-2.337	0.256	0.837
Race	White vs Black	Topic 4	0.2254	0.2805	1.253	0.723-2.171	0.422	0.837
Race	White vs Black	Topic 5	-0.3216	0.3171	0.725	0.389-1.35	0.31	0.837
Race	White vs Black	Topic 6	0.0467	0.2553	1.048	0.635-1.728	0.855	0.855
Race	White vs Black	Topic 7	0.1717	0.2609	1.187	0.712-1.98	0.51	0.837
Stage	Late vs Early	Topic 1	-0.0982	0.2734	0.906	0.53-1.549	0.72	0.832
Stage	Late vs Early	Topic 2	-0.0968	0.2481	0.908	0.558-1.476	0.696	0.832
Stage	Late vs Early	Topic 3	0.1139	0.2795	1.121	0.648-1.938	0.684	0.832

Stage	Late vs Early	Topic 4	-0.1403	0.2846	0.869	0.498-1.518	0.622	0.832
Stage	Late vs Early	Topic 5	0.0668	0.3149	1.069	0.577-1.982	0.832	0.832
Stage	Late vs Early	Topic 6	-0.2277	0.2582	0.796	0.48-1.321	0.378	0.832
Stage	Late vs Early	Topic 7	-0.0805	0.2636	0.923	0.55-1.547	0.76	0.832
Histology	Type II vs Type I/Other	Topic 1	0.196	0.2659	1.217	0.722-2.049	0.461	0.807
Histology	Type II vs Type I/Other	Topic 2	-0.0186	0.2415	0.982	0.611-1.576	0.939	0.939
Histology	Type II vs Type I/Other	Topic 3	-0.0334	0.2719	0.967	0.568-1.648	0.902	0.939
Histology	Type II vs Type I/Other	Topic 4	0.2379	0.2772	1.269	0.737-2.184	0.391	0.807
Histology	Type II vs Type I/Other	Topic 5	-0.2439	0.3069	0.784	0.429-1.43	0.427	0.807
Histology	Type II vs Type I/Other	Topic 6	0.0237	0.251	1.024	0.626-1.675	0.925	0.939
Histology	Type II vs Type I/Other	Topic 7	0.3947	0.257	1.484	0.897-2.456	0.125	0.807

Supplemental Table S3. Complete Statistical Testing Results for *Lactobacillus* Species Abundance

**Clinical Variable**	**Comparison Groups**	**Species**	**Raw p-value**	**FDR q-value**	**Test Method**
Age	<50 vs ≥50 years	*L. crispatus*	0.062	0.125	Wilcoxon
		L. jensenii	**0.034**	0.125	Wilcoxon
		L. iners	0.340	0.454	Wilcoxon
		L. gasseri	0.901	0.901	Wilcoxon
Stage	Early vs Late	*L. crispatus*	**0.017**	0.067	Wilcoxon
		L. iners	0.282	0.564	Wilcoxon
		L. jensenii	0.568	0.758	Wilcoxon
		L. gasseri	0.962	0.962	Wilcoxon
Race	Black vs White	*L. iners*	0.074	0.295	Wilcoxon
		L. crispatus	0.229	0.458	Wilcoxon
		L. gasseri	0.520	0.693	Wilcoxon
		L. jensenii	0.883	0.883	Wilcoxon
Histology	Type I/Other vs Type II	*L. crispatus*	0.071	0.284	Wilcoxon
		L. gasseri	0.555	0.630	Wilcoxon
		L. iners	0.616	0.630	Wilcoxon
		L. jensenii	0.630	0.630	Wilcoxon

Supplemental Table S4. Zero-Inflated and Differential Abundance Sensitivity Analysis of Key Findings

Feature	Level	Primary Comparison	Original Effect (Primary Analysis)	Original FDR q	ZI/DA Method	logFC / Coef	ZI/DA FDR q	Hurdle PA OR (95% CI)	Hurdle PA FDR q	Hurdle CA OR (95% CI)	Hurdle CA FDR q	Direction Consistent?
ASV54 (Actinomycetaceae)	ASV	Race: Black vs White	5.0-fold higher prevalence; 40.9% vs 8.2%	0.005	fitZig (CSS-normalized)	4.18	0.00014	8.69 (2.60–29.10)	0.0023	0.99 (0.43–2.29)	0.97	**Yes**
Actinotignum	Genus	Age: per decade	Higher prevalence ≥50 vs <50 yrs; 68.2% vs 23.8%	0.007	MaAsLin2 (log rel. abundance)	1.43	0.0021	2.37 (1.55–3.64)	0.00035	1.10 (0.91–1.34)	0.39	**Yes**
Cutibacterium	Genus	Stage: Late vs Early	Higher prevalence early vs late stage; 61.0% vs 33.0%	0.048	MaAsLin2 (log rel. abundance)	−1.06	0.55	0.41 (0.15–1.13)	0.42	0.96 (0.80–1.16)	0.84	**Yes***
Peptoniphilus (Topic 4 component)	Genus	Age: per decade	Topic 4 OR = 1.35/decade (95% CI: 1.11–1.63)	0.019	MaAsLin2 (log rel. abundance)	0.78	0.28	1.27 (0.69–2.36)	0.55	1.24 (1.06–1.44)	0.033	**Yes**
Anaerococcus (Topic 4 component)	Genus	Age: per decade	Topic 4 OR = 1.35/decade (95% CI: 1.11–1.63)	0.019	MaAsLin2 (log rel. abundance)	1.17	0.084	1.49 (0.89–2.48)	0.32	1.23 (1.04–1.46)	0.033	**Yes**

Abbreviations: PA = Presence/Absence; CA = Conditional Abundance; FDR = Benjamini-Hochberg false discovery rate; OR = odds ratio; logFC/Coef = log2 fold-change (fitZig) or log coefficient (MaAsLin2); CI = 95% confidence interval. Reference levels: Race = White; Stage = Early.
