# Supplementary material for: Characterizing the composition and diversity of the vaginal microbiome in ovarian cancer
Source: Commun Med (Lond). 2026 Jul 10;6:573. doi: 10.1038/s43856-026-01771-8 (PMC13728130; doi:10.1038/s43856-026-01771-8)
Supplement: Supplementary file 2 [file 43856_2026_1771_MOESM2_ESM.pdf]

Description of Additional Supplementary Files

File name: Supplementary Data 1

Description: The source data behind the graphs in the paper
